# Patient stratification for determining optimal second and third line therapy for type 2 diabetes: the TriMaster study

**DOI:** 10.1038/s41591-022-02120-7

**Published:** 2022-12-07

**Authors:** Beverley M Shields, John M Dennis, Catherine D Angwin, Fiona Warren, William E Henley, Andrew J Farmer, Naveed Sattar, Rury R Holman, Angus G Jones, Ewan R Pearson, Andrew T Hattersley, TriMaster Study group, TriMaster Central Coordinating Team:, Catherine Angwin, Caroline Jenkinson, Nina Rickards, Claire Thorne, TriMaster Study Group, Andrew Hattersley, Catherine Angwin, Claire Ball, Anna Barnes, Tamika Chapter, Daniela Carmona, Tim Cranston, Clare Davidson, Mary Davis, Evan Davy, Tim Eames, Joanne Findlay, Diane Jarvis, Caroline Jenkinson, Angus Jones, John Kirkwood, Bethan Knight, Bridget Knight, James Leavy, Pattie Liakos, Kelly Littlewood, Timothy McDonald, Dionne McGill, Richard Oram, Nicola Pamphilon, Kashap Patel, Andrew Pitt, Lynne Quinn, Shelley Rhodes, Nina Rickards, Emma Robjohns, Kim Rowden, Sofia Sanabria, Beverley Shields, Steven Spaull, Sarah Statton, Anna Steele, Nick Thomas, Claire Thorne, Shirley Todd, Harry Tripp, Robert Wells, Luke Weymouth, Fiona Walters, Ewan Pearson, Gill Reekie, Charlene Wong, Naveed Sattar, Josephine Conney, Robert Lyndsay, Kirsty McLeish, Janice Richardson, Rob Andrews, Ian Bodger, Richard Burgess, Sue Crouch, Isy Douek, Amanda Groves, Catherine Lane, Claire Lorimer, Joy Rowe, Lyndsay Stone, Ali Chakera, Zdenka Cipinova, Zhengmai He, Allison Leslis, Dominika Wlazly, Louise Overend, Sushil Kumar, Joanne Rafferty, Gillian Webster, Rustam Rea, Lia Anguelova Karyna Gibbons, Nicky McRobert, Ursula Taylor, Simon Heller, Mark Davy, Jackie Elliot, Rajiv Ghandi, Sue Hudson, Linda Greaves, Chloe Husband, Peter Novodorsky, Helena Renberg-Fawcet, Kim Ryalls, Lisa Zeidan, Mark Walker, Ahmad AbuSaleh, Jade Davison, Margaret Fearby, Louise Finlay, Donna McEvoy, Ian McVittie, Bijal Patel, Geraldine Richel, l Rebecca Wilson, Thomas Hugh Jones, Mishell Cunningham, Allison Daniels, Laura Walker, Lisa Zeidan, Andrew Johnson, Georgina Russell, Jade Bennett, Shenede Coppin, Joanne Davies, Sharon Hook, Abby Hookey, Jade King, Sharon Merritt, Helen Pearson, Sharon Tovey, Jill Townley, Patrick English, Migaila Aldred, Emma Bishop, Emma Storr, Sam Rice, Kim Davies, Rachel Davies, Linda O’Brien, Zohra Omar, Stephen Bain, Lucy Barlow, Steven Creely, Duncan Browne, Terri Chant, Helen Chenoweth, Kelly Hutchins, Laura Jones, Haider Khan, Emily Laity, Hemanth Bolusani, Adele Farrugia, Grace Hopkins, Emma Norling, Caroline Robinson, Kennedy Cruickshank, Krzystof Rutkowski, Benjamin Field, James Clark, Ed Combes, Ruth Habibi, Stonny Joseph, Louise Allen, Tracy Hazleton, Alicia Knight, Janine Musselwhite, Sutapa Ray, Amanda Gillespie, Christine Cassidy, Peter Hammond, Tahir Idrees, Sam Jackson Neil Lauber, Deirdre Maguire, Ayeaye Min, Simon Trickett, Taryn Ward, Annie Williamson, Chitrabhanu Ballav, Nicola Bowers, Sonia Dayal, Flavio Gil Lopes de Sousa, Lisa Jones, Mari Kononen, Ruth Penn, Fabiana Saraiva, El Muhtadi Saeed, Julie Sutton, Gerry Rayman, Helen Atkins, Emma Galloway, Jo Rosier, Debbie Simmonds, Michael Cummings, Katherine Alington Sharon Allard, Sophie Daltry, Christine Hall, Victoria Hunter, Kate Millar, MASTERMIND Consortium (additional members), Michael Weedon, Robert Lindsay, Christopher Jennison, Mark Walker, Kennedy Cruickshank, Salim Janmohamed, Christopher Hyde, Alastair Gray, Stephen Gough, Lauren Rodgers, Timothy McDonald, Olorunsola Agbaje, TriMaster Data Monitoring Committee, David Owens, Kamlesh Khunti, Christopher Weir, TriMaster Trial Steering Committee, Edwin Gale, Polly Bingley, Caroline Padget, David Russell-Jones, Stephen Senn

**Affiliations:** 1 Department of Clinical and Biomedical Sciences, University of Exeter, Exeter, UK; 2 Clinical Trials Unit, University of Exeter Medical School, Exeter, UK; 3 Institute of Health Research, University of Exeter Medical School, Exeter, UK; 4 Nuffield Department of Primary Care Health Sciences, University of Oxford, Oxford, UK; 5 School of Cardiovascular & Metabolic Health, University of Glasgow, Glasgow, UK; 6 Diabetes Trials Unit, Radcliffe Department of Medicine, University of Oxford, Oxford, UK; 7 Population Health & Genomics, School of Medicine, University of Dundee, Dundee, UK

## Abstract

Precision medicine aims to treat an individual based on their clinical characteristics. A differential drug response, critical to using these features for therapy selection, has never been examined directly in type 2 diabetes. We tested two hypotheses: 1) individuals with BMI>30kg/m^2^, compared with BMI ≤30kg/m^2^, have greater glucose lowering with thiazolidinediones than DPP4-inhibitors, and 2) individuals with eGFR 60-90mls/min/1.73m^2^ compared with eGFR >90mls/min/1.73m^2^ have greater glucose lowering with DPP4-inhibitors than SGLT2-inhibitors. The primary endpoint for both hypotheses was the achieved HbA1c difference between strata for the two drugs. 525 people with type 2 diabetes participated in a UK based randomised, double-blind, three-way crossover trial of 16 weeks treatment with each of sitagliptin 100mg/day, canagliflozin 100mg/day and pioglitazone 30mg/day added to metformin alone or metformin plus sulfonylurea. Overall, the achieved HbA1c was similar for the three drugs pioglitazone 59.6 mmol/mol, sitagliptin 60.0 mmol/mol, canagliflozin 60.6 mmol/mol (p=0.2). Participants with BMI>30kg/m^2^, compared with BMI≤30kg/m^2^, had a 2.88 mmol/mol (95% CI 0.98,4.79) lower HbA1c on pioglitazone than on sitagliptin (n=356, P=0.003). Participants with eGFR 60-90mls/min/1.73m^2^, compared with eGFR >90mls/min/1.73m^2^, had a 2.90 mmol/mol (95% CI 1.19,4.61) lower HbA1c on sitagliptin than on canagliflozin (n=342, P=0.001). There were 2201 adverse events reported, and 447/525 (85%) randomised participants experienced an adverse event on at least one of the study drugs. In this precision medicine trial in type 2 diabetes, our findings support the use of simple routinely available clinical measures to identify the drug class most likely to deliver the greatest glycaemic reduction for a given patient. ClinicalTrials.gov registration: NCT02653209; ISRCTN registration12039221..

## Introduction

Precision medicine aims to tailor treatment to an individual based on their clinical characteristics^1^. The most successful examples of precision medicine to date have been in cancer and monogenic disease, where genetic sequencing has indicated molecularly distinct subtypes that could benefit from specific treatment strategies^2,3^. This approach however is not suitable for common polygenic complex diseases, so other strategies are needed.

Type 2 diabetes is an attractive candidate for a precision medicine approach as it is a heterogeneous disease with varying underlying pathophysiology, and there are many different options for glucose-lowering treatment available which have differing mechanisms of action^4^. Identifying clinical characteristics or biomarkers robustly associated with differential treatment responses could allow the targeting of specific glucose-lowering agents to those most likely to benefit.

In the 2022 ADA/EASD international guidelines, the targeting of therapy based on a person’s clinical features is limited^5^. In patients with established atherosclerotic cardiovascular disease, glucagon-like peptide 1 receptor agonists (GLP-1RA) or sodium–glucose cotransporter 2 inhibitors (SGLT2i) are recommended^5^. Patients with either heart failure, or chronic kidney disease are recommended to receive SGLT2i. However, these recommendations apply to only 15-20% of individuals^6^. For most individuals with type 2 diabetes, current guidelines include a broad choice of potential therapies with differentiation between treatment classes based predominantly on costs and side effect profiles, rather than efficacy.

Simple clinical features, such as a person’s sex, surrogate markers of insulin resistance such as BMI and triglycerides, or markers of renal function such as estimated glomerular filtration rate (eGFR), can be used to stratify people with type 2 diabetes into subgroups showing differential responses to glucose lowering therapies^7^. Individuals with obesity have been shown to have a greater glycaemic reduction on thiazolidinediones (TZD), compared with individuals without obesity, whereas a higher BMI is associated with a smaller glycaemic reduction on DPP4-inhibitors (DPP4i)^8,9^. For SGLT2i, which act through inhibiting the active reabsorption of glucose in the proximal tubule, impaired renal function (lower eGFR) is associated with reduced glucose-lowering efficacy^10,11^. In contrast, with some DPP4i, impaired renal function is associated with increased glucose lowering efficacy, likely due to the drug pharmacokinetics where reduced renal clearance can lead to increased plasma DPP4i concentrations^12^. These associations to date have been observed in independent treatment groups in electronic healthcare records and in post-hoc analyses of individual participant data in parallel group randomised controlled trials^8–11^.

The precision medicine approach to using these data-derived strata needs to be tested in a clinical trial. To date, there have been no trials directly examining a precision medicine approach to prescribing in type 2 diabetes. The effectiveness of any stratified approach for choosing between therapies will depend upon the extent to which differential responses can be predicted, and therefore the true test of a precision medicine approach would be to assess the within-person differential responses to therapy.

**Figure 1:**
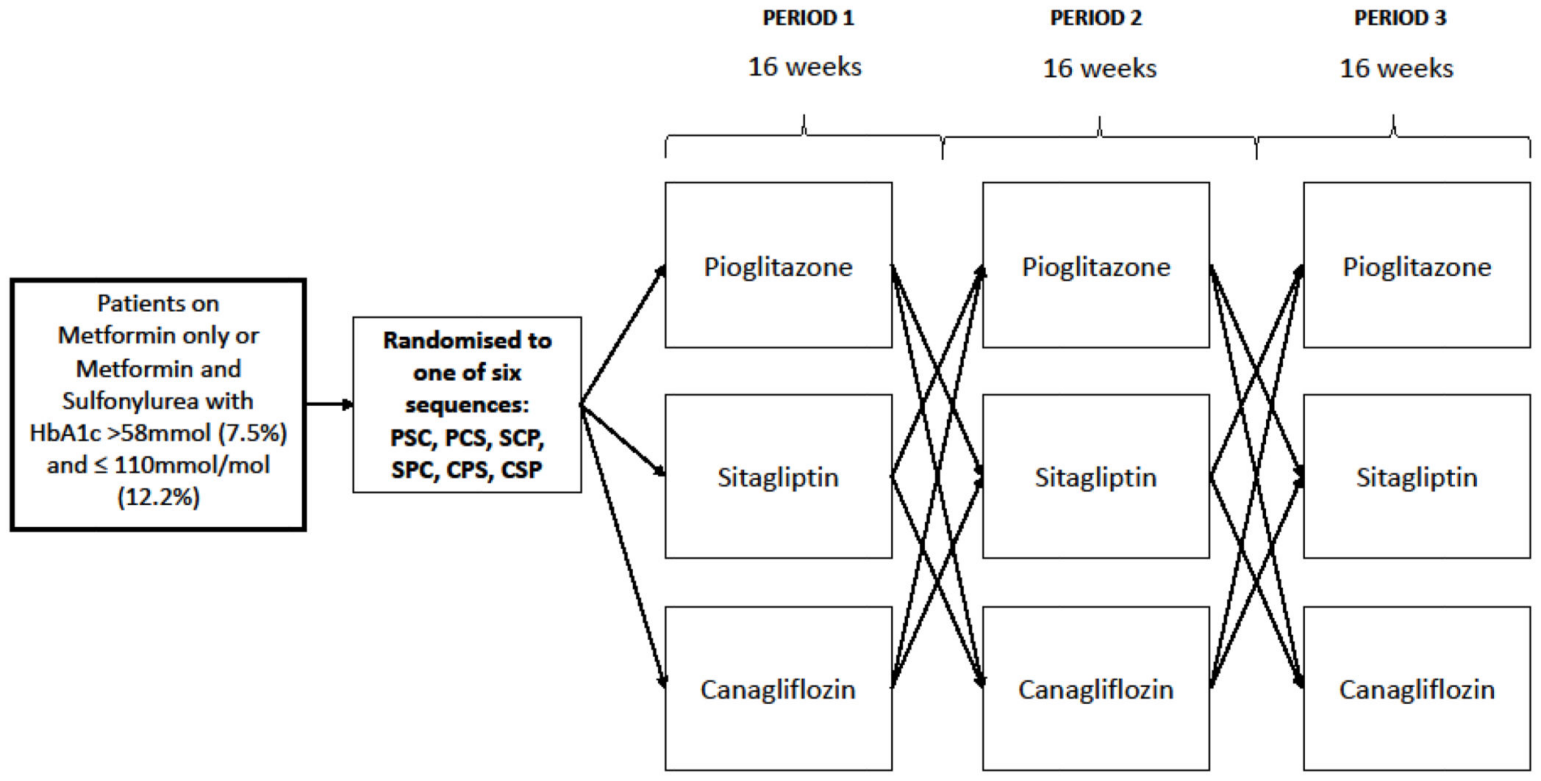
Study design for the TriMaster three-treatment, three-period crossover trial of pioglitazone, sitagliptin, and canagliflozin. Six sequences represent the 6 possible treatment orders for pioglitazone (P), canagliflozin (C) and sitagliptin (S). No washout between treatment periods.

**Figure 2:**
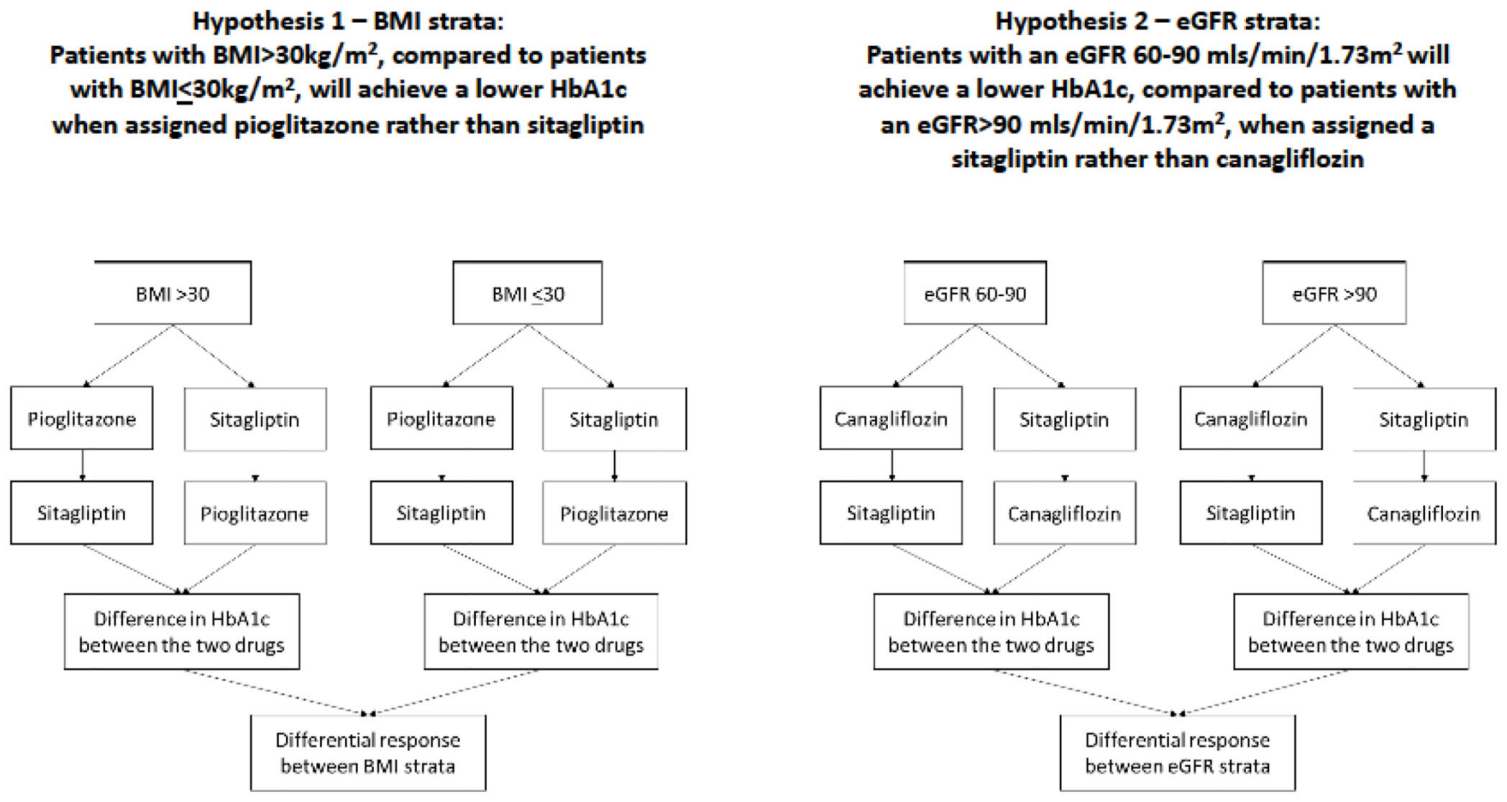
The two main hypotheses being tested in TriMaster

We have carried out a three-drug, three-period, randomised crossover trial to assess two specific hypotheses (Figures 1 and 2), in people with type 2 diabetes treated with metformin alone, or with metformin plus sulfonylurea:

1) Individuals with a BMI >30kg/m^2^, compared with those with a BMI<=30kg/m^2^, will have a greater glycaemic reduction with a TZD (pioglitazone), than with a DPP4i (sitagliptin).
2) Individuals with an eGFR 60-90mls/min/1.73m^2^, compared with those with eGFR>90mls/min/1.73m^2^, will have a greater glycaemic reduction with a DPP4i (sitagliptin), than with an SGLT2i (canagliflozin).

## Results

### Participant retention and baseline characteristics

**Figure 3:**
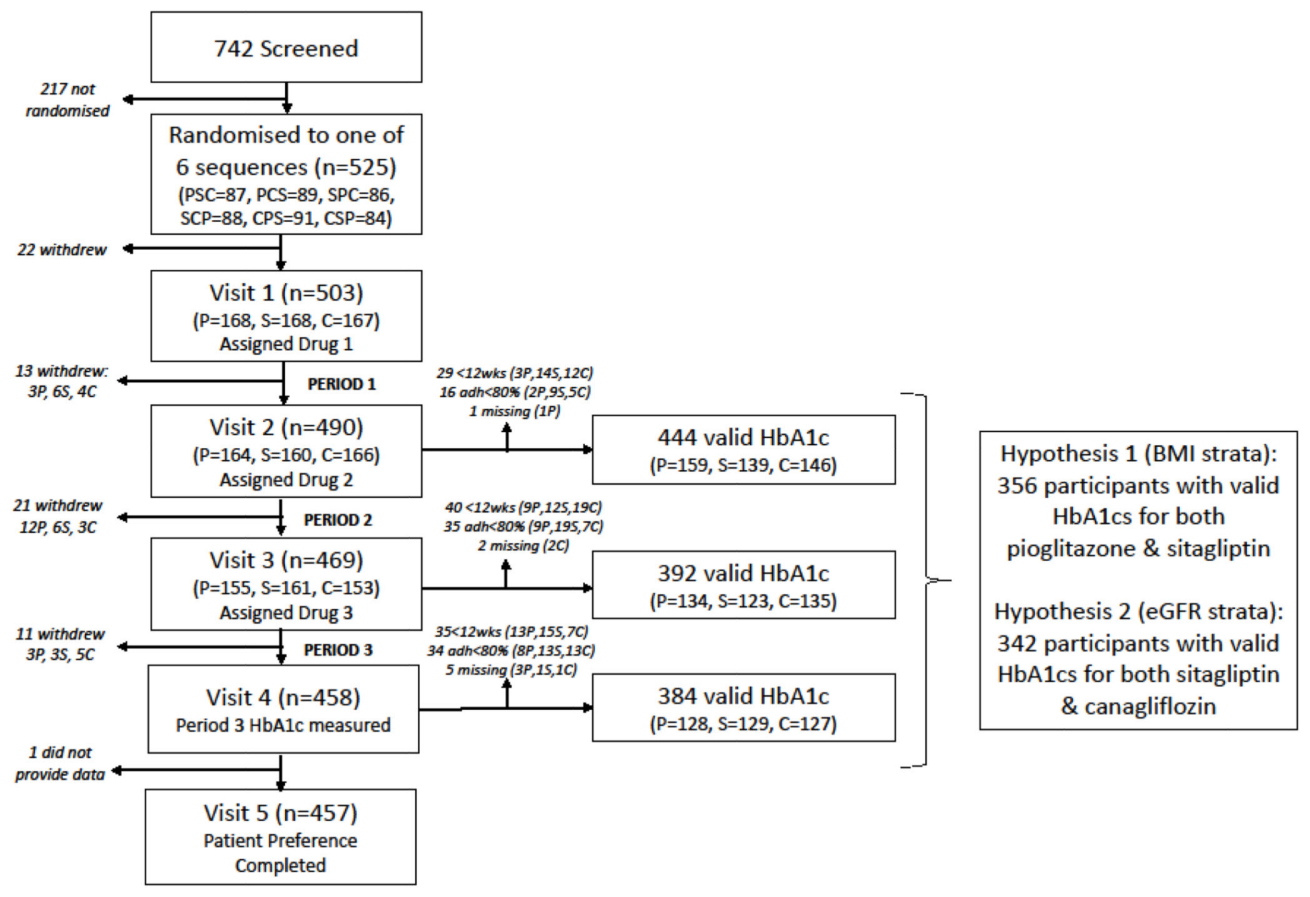
Trial profile (CONSORT diagram): patient flow through the stages of the crossover trial and eligibility for primary analysis. Numbers presented for each visit are the numbers assigned each drug at that time. For an HbA1c to be valid for primary analysis, it needed to be taken after at least 12 weeks of therapy (exclusions indicated by <12wks), and participants needed to have at least 80% adherence on the therapy (exclusions indicated by adh<80%). P= pioglitazone, S=sitagliptin, C=canagliflozin

Figure 3 shows participant flow throughout the study and the numbers on each drug at each stage.

742 patients were screened for eligibility between 22 November 2016 and 24 January 2020. 210 did not meet eligibility criteria and 7 patients withdrew before being randomised. Overall, 525 participants were randomised to one of the six sequences of drug allocations (see Table 1 for participant characteristics). Of these, 20 withdrew prior to the baseline visit (4 health reasons, 10 changed mind, 2 ineligible, 1 moved out of area and 3 unable to contact) and two withdrew at the baseline visit due to difficulties taking blood, leaving 503 receiving their first study drug. Overall, there were 45 participants who subsequently withdrew (Figure 3) leading to 458 participants (87% of those randomised) who completed all 3 study periods. In total, there were 1417 instances of people taking drugs: 469 pioglitazone, 474 sitagliptin, 474 canagliflozin.

For hypothesis 1, 356 participants (68%) had HbA1c results that could be included in primary analysis (i.e. took therapy for at least 12 weeks with >80% adherence based on pill count). For hypothesis 2, 342 participants (65%) had HbA1c results that could be included in primary analysis. No participants were missing eGFR or BMI results.

There was no evidence of any HbA1c carryover effect, but some evidence of a period effect with participants having a mean (95% CI) 1.38 (0.23, 2.54) mmol/mol lower HbA1c in period 2 compared with period 1. There was no difference in period 3 compared with period 1, suggesting this was not a sustained reduction over the year (Supplementary Table 1). Period effect was adjusted for in subsequent analysis.

**Extended Figure 1:**
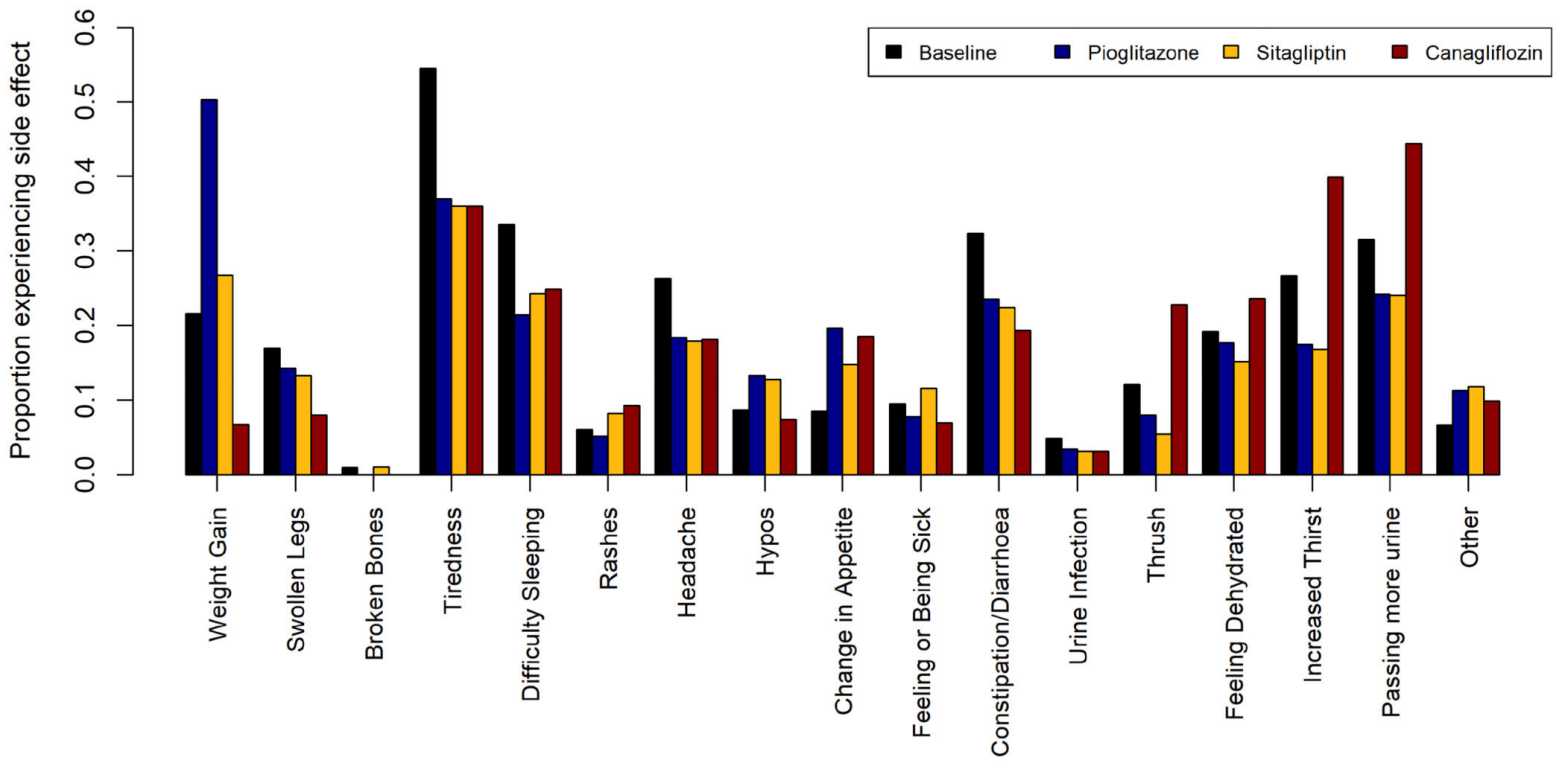
Distribution of side effects experienced on each of the three study drugs (pioglitazone represented by blue bars, sitagliptin by yellow bars, and canagliflozin by red bars) for all instances where people tried the therapy (n=469 pioglitazone, n=474 sitagliptin, n=474 canagliflozin). Proportions experiencing the side effects at baseline shown by black bars.

Prior to stratification, there was no difference in achieved HbA1c between the three therapies pioglitazone 59.6mmol/mol (95% CI 58.5,60.7), sitagliptin 60.0mmol/mol (95% CI 59.0, 61.1), canagliflozin 60.6mmol/mol (95% CI 59.7, 61.6) mmol/mol (p=0.2) (Supplementary Table 2). Pioglitazone was associated with the lowest rates of discontinuation, sitagliptin was associated with the lowest mean number of side effects, and canagliflozin was associated with the lowest weight on therapy (Supplementary Table 2). The distribution of side effects on the three therapies is shown in Extended Figure 1.

### Primary analysis

The five components of the estimand for both hypotheses are shown in Extended Data Table 1.

For hypothesis 1 (BMI dependent differential glycaemic responses to pioglitazone and sitagliptin), 356 (68% of randomised participants) had valid HbA1c values for both pioglitazone and sitagliptin and so were eligible for hypothesis 1 primary analysis (BMI strata). Eligible participants were slightly older and had a slightly lower HbA1c at baseline, compared with those without valid HbA1c values, but were similar with respect to other characteristics (Supplementary Table 3). Characteristics of patients in the two BMI strata are shown in Supplementary Table 4.

**Figure 4:**
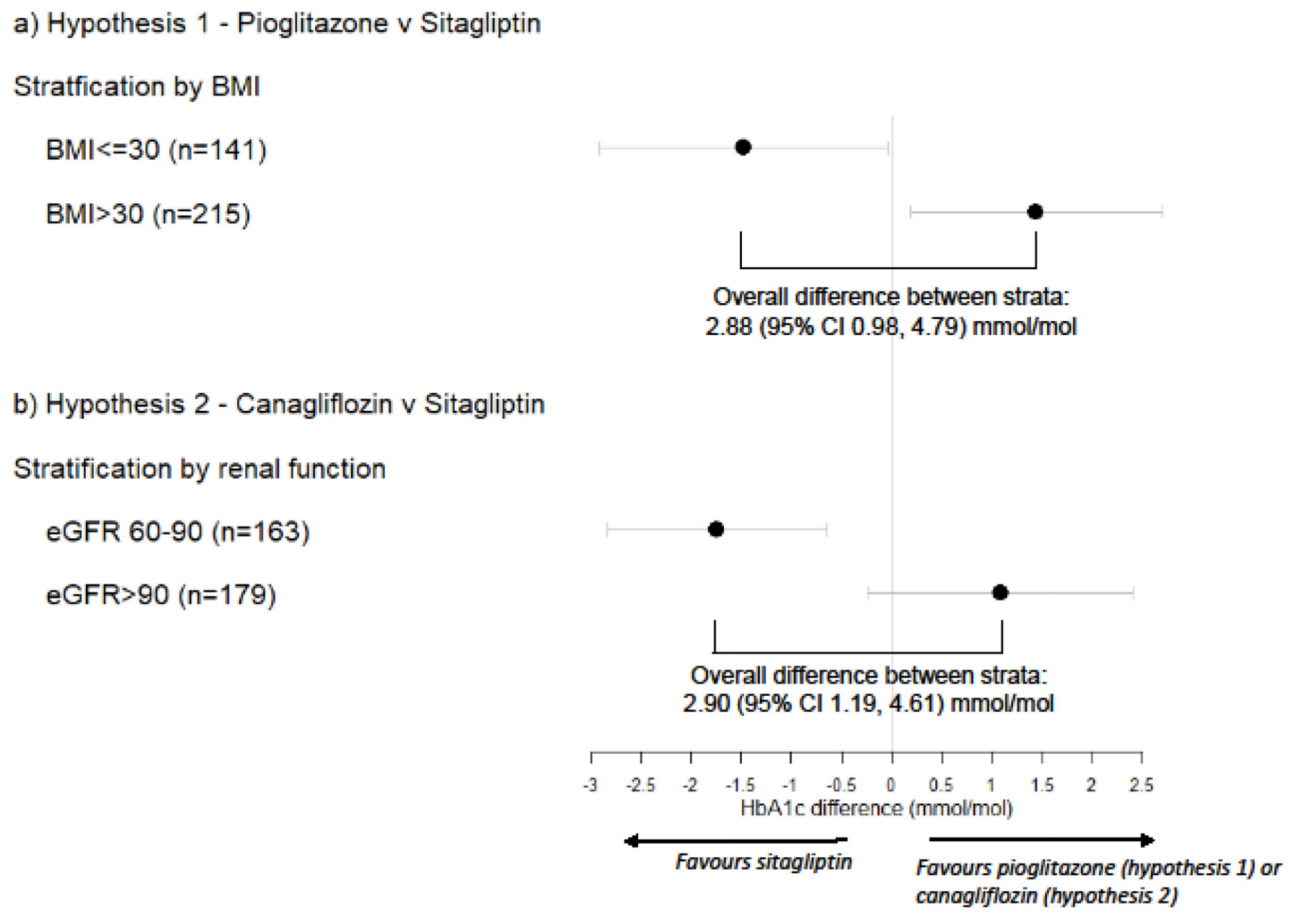
Effect of stratification on treatment response. Point estimates represent the mean difference in HbA1c between the two therapies for a) hypothesis 1 - pioglitazone and sitagliptin, with stratification by obesity (n=141 BMI<=30; n=215 BMI >30), and b) hypothesis 2 – canagliflozin and sitagliptin, with stratification by renal function (eGFR) (n=163 eGFR 60-90; n=179 eGFR>90). Error bars represent 95% confidence intervals. Overall difference between strata determined from drug*strata interaction in mixed effects analysis adjusting for period.

Participants with BMI <=30kg/m^2^ participants had a lower mean 1.48 (95% CI 0.04, 2.91) mmol/mol achieved HbA1c on sitagliptin, compared with pioglitazone. Participants with BMI >30kg/m^2^ had a lower mean 1.44 (0.19, 2.70) mmol/mol achieved HbA1c on pioglitazone, compared with sitagliptin (Figure 4a, Extended Data Table 2). This led to a 2.92 (0.99, 4.85) mmol/mol overall difference between BMI strata. Results were similar in a full mixed effects model, adjusting for period (2.88 (95% CI 0.98, 4.79) mmol/mol, p=0.003) (Supplementary Table 5).

A tipping point analysis suggested the missing data would need to show a 3.1mmol/mol difference in HbA1c in the opposite direction to the trial results to change the statistical significance of the findings.

**Extended Figure 2:**
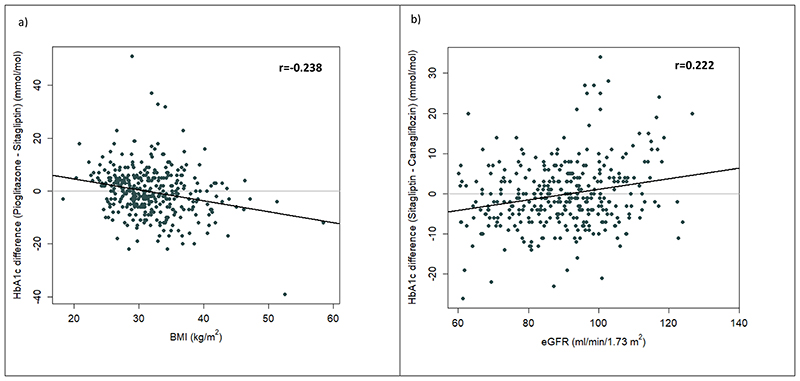
Scatterplots showing a) difference in on-treatment HbA1c between pioglitazone and sitagliptin (negative values favour pioglitazone, positive values favour sitagliptin) against BMI, and b) difference in on-treatment HbA1c between sitagliptin and canagliflozin (negative values favour sitagliptin, positive values favour canagliflozin) against eGFR. Line of best fit shown for each plot.

The association between BMI and difference in response between pioglitazone and sitagliptin was linear on a continuous scale indicating that there would be an even greater benefit for pioglitazone at higher BMIs and greater benefit for sitagliptin at lower BMIs (Extended Figure 2a).

For hypothesis 2 (eGFR and differential responses to sitagliptin and canagliflozin), 342 (65% of randomised participants) had valid HbA1c values for both sitagliptin and canagliflozin, and so were eligible for primary analysis for hypothesis 2 (eGFR strata). There were no differences in characteristics between those eligible and ineligible for hypothesis 2 analysis (Supplementary Table 6). Characteristics of patients in the two eGFR strata are shown in Supplementary Table 7.

Participants with an eGFR 60-90mls/min/1.73m^2^ had a lower mean (95% CI) 1.74 (0.65, 2.85) mmol/mol achieved HbA1c on sitagliptin, compared with canagliflozin. Participants with an eGFR >90mls/min/1.73m^2^ had a lower mean 1.08 (-0.24, 2.41) mmol/mol achieved HbA1c on canagliflozin, compared with sitagliptin (Figure 4b, Extended Data Table 3). In a full mixed effects model, adjusting for period, this translated into a difference of 2.90 (1.19, 4.61) mmol/mol between eGFR strata (p=0.001) (Supplementary Table 8).

A tipping point analysis suggested the missing data would need to show a 3.2mmol/mol difference in HbA1c in the opposite direction to the trial results to change the statistical significance of the findings.

The association between eGFR and difference in response between sitagliptin and canagliflozin was linear on a continuous scale indicating that there would be an even greater benefit for canagliflozin at higher eGFR values and greater benefit for sitagliptin at lower eGFR values (Extended Figure 2b)

Sensitivity analyses show that results did not differ for either of the tested hypotheses when adjusting for study period, when restricted to only those with HbA1c values when on therapy for at least 15 weeks, when adjusting for differences in time intervals between measurements, or when adjusting for those who had >18 weeks of therapy (Supplementary Tables 9-11).

#### Secondary outcomes

There was no difference in tolerability between BMI strata for pioglitazone, compared with sitagliptin (odds ratio (OR) (95% CI) 2.11 (0.66, 6.76) for drug*BMI strata interaction in a mixed effects logistic regression analysis (p=0.2; Supplementary Tables 12 & 13, Extended Data Table 4) or between eGFR strata for canagliflozin compared with sitagliptin (OR (95% CI) 0.424 (0.158, 1.135) for drug*eGFR strata interaction in a mixed effects logistic regression analysis (p=0.09; Supplementary Tables 13 & 14, Extended Data Table 4).

There was no difference in the odds of experiencing at least one side effect for either of the drug/strata combinations of interest (OR (95%CI) 0.68 (0.31, 1.45), p=0.3 for drug*BMI strata interaction; OR (95%CI) 1.46 (0.70, 3.04), p=0.3 for drug*eGFR strata interaction) (Extended Data Table 5, Supplementary Tables 15 & 16).

There was evidence of period and carryover effects for weight, with participants being heavier on average as the trial progressed and with a carryover effect (p<0.001) with either canagliflozin or sitagliptin treatment in the previous period associated with lower weight, compared with pioglitazone treatment in the previous period (Supplementary Table 17). This means absolute weight differences observed between drugs need to be treated with caution. When analysing by strata, pioglitazone was associated with a higher weight compared with sitagliptin in both BMI categories, and this was more pronounced in those with a BMI>30kg/m^2^. (Extended Data Table 6). There was no difference in weight between eGFR strata for canagliflozin and sitagliptin (Extended Data Table 6).

There was no evidence of any difference in the odds of experiencing hypoglycaemia by BMI strata for pioglitazone and sitagliptin, or by eGFR strata for sitagliptin and canagliflozin (Extended Data Table 7, Supplementary Tables 18 & 19).

Participant drug preference was a prespecified secondary analysis and is reported in a separate publication^13^ There was no difference in drug preference by strata. Pioglitazone was ranked higher than sitagliptin in 131/265 (49%) participants in the BMI>30kg/m^2^ strata compared with 78/183 (43%) in the BMI<30kg/m^2^ strata (p=0.2; 10 participants expressed no preference). Sitagliptin was ranked higher than canagliflozin in 112/214 (52%) in the eGFR 60-90 strata compared with 105/235 (45%) in the eGFR>90 strata (p=0.1; 9 participants expressed no preference).

### Adverse events

There were 2201 adverse events reported throughout the study: 56 pre-trial, 1 post-trial and 2144 whilst on therapy in the trial. Table 2 summarises the adverse events on therapy reported throughout the trial. 447/525 (85%) randomised participants experienced adverse events on at least one of the study drugs. 45 events were classed as serious (3 participants died), but none of these were related to the study drugs.

## Discussion

This randomised crossover study provides prospective trial evidence to directly support a stratified approach for therapy to manage glycaemia in type 2 diabetes. Our results demonstrate that for second- and third-line therapy in type 2 diabetes, simple predefined stratification using body mass index and renal function can determine the choice of the drug most likely to be effective for glucose-lowering.

We have shown, among patients with type 2 diabetes on background metformin or combination metformin and sulfonylurea therapy, stratification based on BMI and eGFR is associated with differential glucose-lowering responses to canagliflozin, sitagliptin, and pioglitazone. For a population of people with type 2 diabetes, treating patients with the drug proposed best for their strata rather than the alternative drug could potentially lead to an overall mean improvement of ~3mmol/mol, in those who are able to tolerate the therapy. This stratification could be used to help select glucose-lowering therapies for individuals in clinical practice. For participants with a BMI>30kg/m^2^, a lower HbA1c was achieved on pioglitazone compared with sitagliptin, whereas for those with a BMI<30kg/m^2^, a lower HbA1c was achieved with sitagliptin. For participants with impaired renal function (eGFR between 60 and 90 ml/min/1.73m^2^), a lower HbA1c was achieved on sitagliptin compared with canagliflozin, whereas for those with normal renal function (eGFR>90), a lower HbA1c was achieved on canagliflozin. These findings are concordant with our original study hypothesis. There was no evidence by strata in reported drug tolerability or overall rates of side effects.

We found using different strata led to clinically meaningful differences (~3mmol/mol) in achieved HbA1c between glucose lowering therapies. This equates to approximately 3 years without requiring additional therapy, given the median deterioration in HbA1c in people with type 2 diabetes is 1mmol/mol per year^14^. In contrast, without stratification, all three therapies were on average equivalent in achieved HbA1c. Although, these differences are of a smaller magnitude than the benefits seen with targeted therapy in monogenic diabetes (e.g. a ~30mmol/mol difference in response between metformin and gliclazide treatment for patients with HNF1A_MODY^15^), the overall improvement through stratification would likely have a pronounced effect at the population level as type 2 diabetes is far more common (90% of all diabetes for type 2 compared with <0.5% for MODY)^16,17^. A lack of difference in tolerability or overall incidence of side effects between strata suggests that if choice of therapy were to be based solely on the optimal strata for glycaemic response this would not likely lead to any overall increase in these detrimental effects. However, consideration would need to be made regarding the weight gain associated with pioglitazone, which was greater in individuals with obesity and would need to be balanced against the greater HbA1c improvement. Further work is needed to determine the effect of this on other non-glycaemic effects such as blood pressure.

These findings, based on binary, free-to-implement strata, establish the principle of stratification helping to target type 2 diabetes treatment to those most likely to benefit, and represent a step forward in the translation of type 2 diabetes precision medicine into clinical practice. However, ultimately a more sophisticated ‘precision’ approach using models that integrate multiple individual-level clinical features (eg BMI, HbA1c and eGFR) on a continuous scale will have greatest utility for clinical practice^7,18^. Using individual-level features will likely enable the identification of more ‘extreme’ patient phenotypes with large differences in HbA1c reduction than we demonstrated with binary strata based on clinically defined cut-offs. For example, when we look at the impact of BMI or renal function on a continuous scale rather than two dichotomous groups, it is clear that those with more extreme values have greater differential response to the treatment. Such models could potentially be optimised to incorporate more advanced biomarkers and genetics, and to evaluate additional outcomes beyond HbA1c^19^.

Our findings are consistent with previous research from trial subgroup and observational data which has suggested that higher BMI may be associated with increased glucose lowering to thiazolidinediones^9^ and modestly reduced glucose lowering to DPP4-inhibtors^8^, and with research suggesting that lower eGFR is associated with reduced response to SGLT2-inhibitors^10,11^. Pioglitazone acts through altering the transcription of genes influencing carbohydrate and lipid metabolism in adipocytes^20^, which could lead to a greater glycaemic effect in those with higher BMI. For sitagliptin, which reduces degradation of incretin hormones, including GLP-1, thereby potentiating insulin secretion, the association of greater HbA1c reduction in those with a lower BMI is less clear. Potential mechanisms include the impact of high insulin resistance on the action of a drug which acts predominantly through potentiating insulin secretion, impaired GLP-1 secretion in obesity, or direct effects of lipotoxicity on GLP-1 receptor expression which have been demonstrated in animal models^21–23^. For SGLT2i,the drug mechanism of action to lower glucose levels per se (as opposed to its other effects) is through inhibition of renal tubular glucose reabsorption, and a low eGFR might therefore be expected to lead to reduced filtration of glucose, and subsequently reduced glycosuria with SGLT2i therapy^24^.

A key strength of this randomised controlled trial (RCT) is that we have shown that these differences are observed in the crossover setting, allowing robust assessment of differential response to therapy within individuals and therefore direct assessment of stratified treatment that cannot be undertaken from existing trials with a parallel group design. The crossover design also requires a much smaller sample size compared with parallel group trials. There were a number of limitations to our RCT. The crossover design, although more powerful for assessing within-person differences, does require careful design to avoid period and carryover effects. We did see a period effect with a reduction in HbA1c in the second period, but in line with our statistical analysis plan we adjusted for this in our analysis, and this was not a sustained reduction over the year, which would indicate a more general decline in glycaemic control. We did not see a carryover effect for HbA1c, our primary outcome, but there was carryover with weight limiting the interpretability of the effect sizes for the associations seen with weight. In addition, the crossover design only enabled an assessment of short-term outcomes, meaning that we did not evaluate durability of HbA1c reduction, cardiovascular outcomes or development of diabetes complications. However, our previous work using parallel group trial data and observational data suggest that differences in response associated with strata are maintained over time, with early HbA1c response representative of long-term effects^8,25^. The majority of our study population were male (73%) and self-reported white ethnicity (94%), which limits conclusions about the relative benefits and risks of these therapies in females and in other ethnic groups. We assessed only specific glucose-lowering agents and findings cannot be assumed to reflect class effects of SGLT2-inhibitors, DPP4-inhibitors, and thiazolidinediones. We chose a perprotocol analysis rather than intention-to-treat approach for our primary analysis as we could not obtain a valid HbA1c value when participants had not taken the therapy for at least 12 weeks, and imputation with baseline measures was deemed inappropriate due to the pre-study baseline not being representative for later study periods. This means the inferences from this study apply only to those who can tolerate the therapies of interest. There were some minor differences between individuals included and excluded from the BMI-defined strata (hypothesis 1), but there was no difference in tolerability between the study drug and/or strata combinations. In addition, tipping point analysis indicated that the missing data would have to show large differences in the opposite direction to change the statistical significance of our findings. Therefore, we are confident that our findings are not artefacts of our analytical approach and are reflective of the effects seen in those who are able to tolerate the respective therapies.

It should be recognised that we have only studied patients treated with metformin (with or without a sulphonylurea) at baseline and that the glycaemia and tolerability related outcomes we have studied are not the only factors considered by clinicians and patients when choosing a glucose lowering therapy for a patient with type 2 diabetes. In patients with established atherosclerotic cardiovascular disease (or those at elevated risk), or chronic kidney disease or heart failure, SGLT2-inhibitors are the recommended drugs in international guidelines, and GLP1-RAs are recommended for those with atherosclerotic cardiovascular disease^26^. In addition, despite still being a low-cost treatment option proposed in guidelines, prescribing of thiazolidinediones is declining^27,28^. Any precision medicine approach based on short-term outcomes such as glycaemia will need to be embedded in existing treatment pathways based on the longer term cardiorenal risk benefits of specific therapies. In patients without specific cardiorenal indications (~80% of patients^6^) the 2022 ADA/EASD updated guidelines offer many treatment options, so considering likely glycaemic response (based on participant characteristics), alongside other factors considered in current practice (such as cost, and side effect profile) may offer a low cost approach to improving treatment response and patient outcomes.

We have shown, in a randomised crossover study, that clinically relevant differences in glycaemic responses to therapy in type 2 diabetes can be seen when stratifying a patient population based on BMI and eGFR, leading to benefits in those who tolerate these therapies that would not be observed if considering overall glycaemic response to the three drugs in the population as a whole. This study represents a prospective demonstration of a potential stratified approach to type 2 diabetes treatment.

## Methods

### Ethics

The study was approved by the UK Health Research Authority Research Ethics Committee South Central—Oxford A (16/SC/0147).

This trial was conducted and analysed in line with the previously published protocol^29^ and the statistical analysis plan (The full TriMaster Statistical Analysis Plan is freely available and can be downloaded from https://ore.exeter.ac.uk/repository/handle/10871/125162). The trial was registered at ClinicalTrials.gov (NCT02653209) and the ISRCTN registry (12039221). Major protocol amendments were approved by the Royal Devon University Healthcare NHS Foundation Trust as Sponsor, UK Health Research Authority (HRA) Research Ethics Committee South Central—Oxford A and the UK Medicines and Healthcare products Regulatory Agency (where relevant). Details of all 12 major amendments are included in Extended Data Table 7: Protocol Amendments in the TriMaster randomised three-way crossover trial.

### Study design

We conducted a double blind, randomised crossover trial of three glucose-lowering therapies (pioglitazone 30mg once-daily, sitagliptin 100mg once-daily, and canagliflozin 100mg once-daily) in 24 UK centres (Supplementary Table 20). The three-way crossover trial was undertaken as an efficient, faster and more cost-effective approach to address both hypotheses, requiring fewer participants than performing two 2-way crossover studies. In addition, this study design allows a unique opportunity to compare the effects of these 3 medications within a single person, including participant tolerance and therapy preference^13^

### Study participants

Participants were adults aged ≥30 and ≤80 years, with a clinical diagnosis of type 2 diabetes for at least 12 months and treated with either metformin alone or two classes of oral glucose-lowering therapy (given either as separate or combined medications), that do not include a DPP4-inhibitor, a SGLT2-inhibitor or a thiazolidinedione. This was likely to be metformin and sulphonylurea but included prandial glucose regulators nateglinide or repaglinide. No change of diabetes treatment (new therapy or dose change) was permitted in the previous 3 months. Participants had an HbA1c > 58mmol/mol (7.5%) and ≤110mmol/mol (12.2%) and eGFR ≥ 60mls/min/1.73m^2^, both results confirmed at a screening visit, and were able and willing to give informed consent

Patients were excluded if screening blood tests identified alanine transaminase (ALT) >2.5 x upper limit of the assay normal range (ULN) or known liver disease, specifically bilirubin >30 μmol/L associated with other evidence of liver failure, an HbA1c ≤58mmol/mol (7.5%) or >110mmol/mol (12.2%), or eGFR <60mls/min/1.73^2^. Treatment with insulin in the previous 12 months, or any of the study drugs within the previous 3 months was an exclusion criteria, as was current treatment with corticosteroids, rifampicin, gemfibrozil, phenytoin and carbamazepine, loop diuretics (furosemide or bumetanide) or antibiotics for active infection. Presence of limb ischaemia shown by absence of both pulses in one or both feet at screening, a foot ulcer requiring antibiotics in the previous 3 months or any active infection requiring antibiotics were exclusions.

Patients could not be recruited if undergoing current/ ongoing investigation for macroscopic haematuria, had recent (within 3 months) or planned significant surgery, or had experienced an acute cardiovascular episode (angina, myocardial infarction, stroke, transient ischemic episode) within the previous 3 months. Also excluded were patients with any history of heart failure, bladder carcinoma, diabetic ketoacidosis or pancreatitis. Patients were not recruited if pregnant, breastfeeding or planning a pregnancy over the study period, and concurrent participation on another clinical trial of investigational medicinal product (CTIMP) where the investigational medicinal product (IMP) was currently being taken, without sufficient washout period (5x half-life of IMP/potential-IMP) was also not permitted.

Participants were identified in primary care and from existing research cohorts. People with type 2 diabetes were eligible if aged 30-80 years on stable doses of metformin alone, or metformin plus a sulfonylurea, with HbA1c >58mmol/mol (>7.5%) and <110mmol/mol (≥12.2%). Figure 1 shows the design of the trial. Participants provided written informed consent. Ethnicity was self-reported by participants against standard 2011 UK Office for National Statistics (ONS) coding. Those meeting screening criteria and consenting to take part were randomised to one of the 6 possible therapy sequences and asked to take each allocated therapy in turn for 16 weeks, with both participant and investigators blinded to therapy allocation. There was no washout between therapies. The 16-week treatment period was designed to minimise any carryover (the effects of the previous treatment on the HbA1c in the subsequent period): all three drugs have half-lives between 7 and 14 hours, and HbA1c measurement reflects the previous 8 to 12 weeks of glycaemia. Therefore, the end of treatment period HbA1c represented the initial glycaemic response to the drug for that individual.

### Randomisation and blinding

Randomisation was carried out at the baseline visit as described in the study protocol and statistical analysis plan. The three therapies were allocated in random order according to six possible treatment orders: ABC, ACB, BAC, BCA, CAB, CBA. Drugs were blinded by over-encapsulation (Tayside Pharmaceuticals, Dundee, UK) with allocations blinded to the participants, study team, study researchers, and study statistician.

### Study procedure

Within two weeks of screening, participants attended a baseline fasting visit. Subsequent research visits were scheduled to take place after 16-18 weeks of study treatment, but participants were offered the opportunity to stop a treatment early and move on to the next treatment period if they were unable to tolerate the therapy. At the baseline and end of therapy visits, blood samples were collected for measurement of HbA1c, weight and blood pressure, and the participant’s experiences of the therapy and potential side effects were recorded (once-daily). Participants were compensated for travel expenses only.

### Biochemistry measures

Recruiting centres used local results to confirm eligibility, but all biochemical tests used in analysis, except HbA1c, were centrally analysed at Exeter Clinical Laboratory. These included albumin, aspartate aminotransferase (AST), bilirubin, NT-pro-BNP, cholesterol, C-peptide, creatinine, fructosamine, glucose, HDL-cholesterol, islet autoantibodies (GAD, IA2, ZnT8), insulin, LDL-cholesterol, and triglycerides. To ensure standardisation across centres, eGFR was calculated using the CKD-EPI equation by the central database, based on serum creatinine, sex, ethnicity, and age as collected at baseline. All HbA1c assessment was performed by recruiting centre NHS laboratories to ensure results were available for screening and to inform final patient preference. HbA1c assays were CE marked, fully validated, and accredited by the UK Accreditation Service.

### Measurement of adherence

Participants were asked to return their medication bottle and all unused capsules at the end of the study, with adherence in each treatment period expressed as a percentage calculated as number of tablets taken divided by the expected number of tablets to be taken (number of days between study visits). Where pill count was not available, adherence was based on 4 questions around self-reported compliance (if the patient ever forgot to take their medicine, if they were careless about taking their medicine, if they stopped taking their medicine if they felt unwell, if they stopped taking their medicine if they felt better). Participants were considered to be non-adherent if they answered yes to at least three out of the four questions.

### Outcome measures

#### Primary outcome

The primary outcome was the HbA1c value achieved after each treatment period as long as the participant had taken the study drug for at least 12 weeks and had at least 80% adherence on therapy.

#### Secondary outcomes

The following secondary outcomes were assessed:

1. Tolerability, defined as taking the drug for at least 12 weeks.
2. Participant reported side effects, assessed at the end of each treatment period (see Supplementary Table 21 for full list). For analysis by strata, these were summarised into a binary variable ‘any’ or ‘none’ for each drug for each participant. We defined side effects as any experienced in the treatment periods, including those where they were also reported at baseline.
3. Weight on each therapy, measured at the end of each treatment period.
4. Participant reported experience of hypoglycaemia at the end of each treatment period (binary variable: experienced at least one episode of hypoglycaemia v none). Low blood glucose was defined as either ‘episodes of hypoglycaemia where you felt confused, disorientated or lethargic, and were unable to treat yourself’ or ‘hypoglycaemic episodes where you were unconscious or had a seizure and needed glucagon or intravenous glucose’. At both baseline and subsequent time points, number of episodes, or experience of hypoglycaemia was self-reported and collected on data collection forms.
5. Patient preference of therapy. Participants ranked the three drugs in overall preference, 1 for most preferred, 3 for least preferred.

In line with a change to the statistical analysis plan that we specified prior to data lock, analysis and unblinding, we did not analyse gender differences as a secondary outcome as our study was powered for a 60:40 split in strata, whereas 73% of our cohort were male.

### Adverse event recording

Adverse events or reactions were recorded as they presented or at research visits and reported to the sponsor and Data Monitoring Committee at regular intervals. Adverse events were rated in terms of severity, seriousness, and causality and coded according to MedDRA dictionary terms.

### Changes to protocol

All protocol amendments are detailed in Extended Data Table 8

## Statistical analysis

All analyses were carried out in line with the TriMaster Statistical Analysis Plan, which was signed off prior to data lock and drug allocations being provided. Investigation of participant preference, including additional exploratory analysis, is reported separately (submitted in parallel).

All analysis was carried out using a validated version of Stata v16.1. In line with the SAP, statistical significance was defined as p<0.05, based on two-sided tests of significance.

### The effect of stratification by clinical features

Figure 4 shows the overall approach for the primary analysis for the two hypotheses. For each hypothesis and corresponding drug comparison, the aim was to assess whether the difference in achieved HbA1c for the two drugs differed for the two strata (either BMI above or below 30kg/m^2^, or eGFR above or below 90mls/min/1.73m^2^); the null hypothesis being that the difference in HbA1c between drugs will be the same between strata.

### Rationale for a per protocol approach

Analysis was carried out using a per-protocol approach. For a participant to be included in primary analysis, it was necessary to have a valid HbA1c. For intention to treat analysis, in the absence of a valid HbA1c, some form of imputation of missing values would be required. This is more challenging in a crossover setting, as parallel group approaches such as imputing with the baseline are not valid as the pre-treatment baseline is only an appropriate baseline for the first period. However, we recognise the missing data could be informative. Therefore, to address this issue, we proposed two further analyses to explore the extent to which the missing HbA1cs could affect the final results: a tipping point analysis (see sensitivity analysis) and a secondary analysis of tolerability.

#### Carryover and period effects

Carryover and period effects were checked prior to main analysis. In line with the Statistical Analysis Plan, we examined first order carryover effects (i.e. carryover from the preceding period only) using mixed effects models with drug, period, and a carryover variable (i.e. drug in previous period) as fixed effects, participant as a random effect, and HbA1c as the outcome. The carryover variable used the same coding as the drug variable, or a 0 if in the first period (adjustment for period removes this part of the carryover term in analysis). We adjusted for period in primary analysis by adding as a fixed effect variable in the mixed effects models.

#### Primary analysis

For each hypothesis, the mean (95% confidence interval) for the difference in HbA1c between the two drugs of interest was calculated and also the mean (95% confidence interval) difference of these differences (treatment contrasts) between the two strata of interest (Figure 4). Distribution of HbA1c difference was checked and confirmed to be normally distributed. For the main analysis, a mixed effects model was used for each hypothesis to allow adjustment for study period, with HbA1c as the outcome, participant as the random effect, and drug, period, stratum and drug*stratum interaction as fixed effects. The drug*stratum interaction represented the effect size of interest.

#### Pre-specified sensitivity analyses

1) We examined whether substantial amendment to protocol SA6 (expanding the inclusion criteria to including participants treated with metformin alone, as well as metformin and sulfonylureas) affected the main findings by adding in an “epoch” term to the model, where “epoch” was a binary variable representing before or after the change in inclusion criteria.
2) We repeated the main analysis but including only participants who completed the full treatment period (at least 15 weeks to allow for flexibility in arranging study visits).
3) We examined whether receiving the study drug for >18 weeks (substantial amendment to protocol SA12 in relation to COVID-19 pandemic) affected the main findings, by adding in a binary variable to the model for those with treatment periods greater or less than 18 weeks.

#### Tipping point analysis

As we were analysing using a per-protocol approach, a tipping point analysis was used to explore what change in treatment contrast would be required as a result of the missing data to significantly change the outcome^30^. The tipping point, Δ, was designated according to when it would change the outcome of the study at the 5% significance level, calculated by:

$$\Delta =\frac{\left(1.96*SE\right)-\tau }{f}$$

where τ is the main effect size from analysis (difference in treatment contrasts between stratum), SE is the standard error of this effect size, and f is the fraction of the cohort with missing data.

#### Secondary analyses relating to stratification hypotheses

Tolerability: We tested whether the odds of each of tolerability, side effects (any versus none), and hypoglycaemia (any versus none) differed by the two hypothesised drug/stratum combinations. Each of these secondary outcomes were binary variables, so analysis followed the same approach as primary analysis using the same predictors but using mixed effects logistic regression models instead. As before, the drug*strata interaction represented the effect size of interest but this time the output was an odds ratio, as the data were binary.

Weight: We assessed differences in weight by drug/stratum as in the primary analysis hypotheses using similar mixed effects models to that used in primary analysis but with weight as the outcome.

Patient preference: For each hypothesis, we examined whether patient’s preferred drug differed by strata. All other analysis relating to patient preference is reported in an additional paper submitted separately (reference). For each hypothesis, we compared whether the proportions preferring each of the two drugs of interest differed by strata using the chi-squared test.

#### Secondary analyses of overall differences in outcomes

Overall weight and HbA1c: Mean and SD for weight and HbA1c for each of the three drugs were examined, with statistical differences across all three determined using mixed effects models with drug (3 level factor) as the fixed effect and participant ID as the random effect.

Overall side effects: This analysis was descriptive examining the proportions reporting experiencing each of the 16 side effects the patients were asked about for each of the three drugs.

Overall Tolerability: We report proportions not tolerating therapy (i.e. not completing at least 12 weeks of therapy) for each drug. As specified in the SAP, we compared tolerability using both a Mantel-Haenszel approach and a mixed effects model. Results were similar using both approaches, but for clarity we just present the p values based on the mixed effects model with tolerability as the outcome, drug and period as fixed effects, and participant ID as the random effect.

#### Sample size

For each hypothesis, to detect a difference of 0.35SDs (equivalent to a 3.0mmol/mol difference between the two strata on the two different therapies with 90% power, alpha 0.05, we required 172 participants in each stratum. To allow for the possibility of uneven numbers in each stratum (up to a 60%:40% split), the sample size was increased to 358. To allow for a withdrawal rate of 15% and exclusion from primary analysis due to discontinuing at least one study drug (estimated at 19%), the sample size was increased to 520.

#### Differences to Statistical Analysis Plan

For side effects, in the SAP we stated we would examine only new side effects (i.e. not previously experienced), but changed to any side effects following discussion of presentation of findings. By only examining new side effects, this did not allow us to show change in side effects from baseline. It was apparent that the proportion experiencing some side effects went down on treatments compared with baseline, whereas some went up. By allowing analysis of all we were able to demonstrate this. It also meant participants could record the same side effect on two different drugs, which would not be possible otherwise. The full distribution of participants reporting side effects for baseline and on each of the therapies is presented for completeness.

#### Additional analyses to original Statistical Analysis Plan

There were no major changes to the analysis proposed compared with the original statistical analysis plan, but some minor additional analysis was carried out to explore differences in side effects (any v none) between drugs and strata, as a way of capturing the overall burden of side effects. A strata specific analysis for each side effect would entail multiple testing without prior hypotheses and increased likelihood of type 1 errors, so this was deemed inappropriate.

We also report numbers of adverse events for each drug, split by severity and relatedness and whether they were associated with withdrawal or non-tolerability.

A further sensitivity analysis was conducted to explore the impact of residual autocorrelation arising from time trends or treatment carryover on the main effect sizes (drug/strata interactions). The mixed effects models for the primary analysis were extended by defining an exponential autocorrelation structure for the residual errors. This allowed for the pairwise correlation between HbA1c measurements to decrease systematically as the time gap increased and could account for irregularly spaced intervals.

Finally, we added in scatterplots to show the association on a continuous scale between each of BMI and eGFR against the difference in HbA1c for the two drugs of interest for each hypothesis. We present Pearson correlation coefficients to show the strength of associations alongside these.

#### Missing data

No imputation was carried out and missing data was minimal. Participants required eGFR and BMI to be included in primary analysis, but this was available on all randomised participants. We report n for each analysis and in the tables of results throughout.

## Extended Data

**Extended Data Table 1:** Five components of the estimand for both study hypotheses

Target population	People with type 2 diabetes treated with metformin (+/- sulfonylurea), with HbA1c >58mmol/mol (>7.5%) and <110mmol/mol (<12.2%) indicating treatment with another glucose-lowering therapy is needed.
Treatment condition of interest	Treatment with pioglitazone, sitagliptin, or canagliflozin
Variable of interest	On-treatment HbA1c
Comparative summary measure for results at population level	Difference in HbA1c when treated with the drug proposed best for a particular strata compared with the alternative drug in the other strata.
Handling of intercurrent events	In line with our primary endpoint definition, participants who did not take therapy for sufficient time (discontinued before 12 weeks) or did not take sufficient quantities of therapy (<80% adherence on pill count) were excluded (see methods).

**Extended Data Table 2:** Primary analysis hypothesis 1. Absolute unadjusted values for HbA1c on pioglitazone and sitagliptin split by BMI strata and the corresponding mean difference between drugs, and between strata. P value assessed by a t test comparing the difference between drugs between strata.

BMI category	Drug	HbA1c (mmol/mol) Mean (SD)	Difference between drugs (mmol/mol) Mean (95% CI)*	Difference between strata (mmol/mol) (95% CI)	p
BMI<=30 n=141	Pioglitazone	59.7 (10.4)	1.48 (0.04, 2.91)	2.92 (0.99, 4.85)	0.003
	Sitagliptin	58.3 (8.6)			
BMI>30 n=215	Pioglitazone	59.0 (11.5)	-1.44 (-2.70, -0.19)		
	Sitagliptin	60.5 (11.2)			

* negative values favour pioglitazone.

**Extended Data Table 3:** Primary analysis hypothesis 2. Absolute unadjusted values for HbA1c on sitagliptin and canagliflozin split by eGFR strata and the corresponding mean difference between drugs, and between strata. P value assessed by a t test comparing the difference between drugs between strata.

eGFR category	Drug	HbA1c (mmol/mol) Mean (SD)	Difference between drugs (mmol/mol) Mean (95% CI)*	Difference between strata (mmol/mol) (95% CI)	p
eGFR60-90 (n=163)	Sitagliptin	59.0 (9.6)	-1.74 (-2.85, -0.65)	2.83 (1.09, 4.55)	0.002
	Canagliflozin	60.7 (8.7)			
eGFR>90 (n=179)	Sitagliptin	60.6 (11.7)	1.08 (-0.24, 2.41)		
	Canagliflozin	59.6 (9.4)			

* negative values favour sitagliptin.

**Extended Data Table 4:** Tolerability by Hypothesised Drug/Strata combinations. Proportions tolerating therapy (remaining on therapy for at least 12 weeks) for each of the drug/strata combinations

Hypothesis 1	Pioglitazone	Sitagliptin
BMI>30kg/m^2^	267/277 (96.4%)	255/278 (91.7%)
BMI<=30kg/m^2^	176/192 (91.6%)	175/196 (89.2%)
		
Hypothesis 2	Sitagliptin	Canagliflozin
eGFR 60-90	203/227 (89.4%)	206/221 (93.2%)
eGFR>90	227/247 (91.9%)	227/253 (89.7%)

**Extended Data Table 5:** Side effects by Hypothesised Drug/Strata Combinations. Proportions experiencing at least one side effect for each of the hypothesised drug/strata combinations.

Hypothesis 1	Pioglitazone	Sitagliptin
BMI>30kg/m^2^	238/277 (86%)	217/278 (78%)
BMI<=30kg/m^2^	152/192 (79%)	148/196 (76%)
		
Hypothesis 2	Sitagliptin	Canagliflozin
eGFR 60-90	174/227 (77%)	178/221 (81%)
eGFR>90	191/247 (77%)	215/253 (85%)

**Extended Data Table 6:** Weight difference by drug and strata. P value assessed by a t test comparing the difference between drugs between strata.

		Weight (kg) Mean (SD)	Difference between drugs (mmol/mol) Mean (95% CI)*	Difference between strata (mmol/mol) (95% CI)*	p
BMI category					
BMI<=30 (n=186)	Pioglitazone	80.6 (10.6)	0.97 (0.61, 1.32)	0.93 (0.37, 1.48)	0.001
	Sitagliptin	79.6 (10.6)			
BMI>30 (n=265)	Pioglitazone	104.9 (16.4)	1.89 (1.50, 2.29)		
	Sitagliptin	103.0 (16.4)			
eGFR category					
eGFR 60-90 (n=213)	Sitagliptin	92.6 (16.9)	2.32 (1.95, 2.69)	0.13 (-0.40, 0.66)	0.6
	Canagliflozin	90.3 (16.9)			
eGFR>90 (n=241)	Sitagliptin	93.6 (20.1)	2.19 (1.81, 2.57)		
	Canagliflozin	91.3 (19.9)			

**Extended Data Table 7:** Hypoglycaemia by hypothesised drug/strata combinations. Proportions experiencing hypoglycaemia for each of the hypothesised drug/strata combinations.

Hypothesis 1	Pioglitazone	Sitagliptin
BMI>30kg/m^2^	33/263 (12.5%)	28/272 (10.3%)
BMI<=30kg/m^2^	17/185 (9.2%)	16/182 (8.8%)
		
Hypothesis 2	Sitagliptin	Canagliflozin
eGFR 60-90	17/220 (7.7%)	12/221 (5.4%)
eGFR>90	27/234 (11.5%)	17/246 (6.9%)

**Extended Data Table 8:** Protocol Amendments in the TriMaster randomised three way crossover trial

Amendment no., protocol version, and date	Description
**SA1** v3 06.07.16	Amendment to randomisation process to allocate individual bottles rather than ‘packs’ of 3 bottles to allow for shorter expiry dates, and clarification of safety reporting procedures
**SA4** v4 20.03.17	Amendment to exclusion criteria to allow patients who have previously tried the study drugs to be included, as long as this has not been in the previous 3 months. The original criteria was unnecessarily strict and did not reflect real-world prescribing habits. The amendment also removed the blanket exclusion for patients in concurrent clinical trials, providing sufficient washout period between IMPs.
**SA6** v5 01.08.17	Amendment to eligibility criteria to include patients taking metformin-only, or metformin and a sulfonylurea. This was adjusted due to the change in guidelines and prescribing trends leading to decline in use of sulfonylureas. At the time of study design sulfonylureas were the most commonly prescribed second line therapy in the UK. Subsequent decline in their use in favour of DPP4-inhibitors and SGLT2 inhibitors ^22^, resulted in the inclusion of patients currently treated with either metformin and sulfonylureas or metformin only. We will perform a sensitivity analysis to determine if the difference in study “epoch” (before/after this amendment) has any impact on the main study outcomes. Altered exclusion criteria also added ‘limb ischaemia’ due to updated safety information for Canagliflozin, and an upper limit of HbA1c >110mmol/mol.
**SA9** v6 15.05.18	Amendment to sample size due to over-cautious sample calculations (alpha changed to 0.05), extension to recruitment period due to delays in regulatory approvals at study set-up and slow early recruitment, and additional secondary analysis included on the advice of the Data Monitoring Committee.
**SA10** v7 22.02.19	Amendment to study analysis plan. Following advice from the Trial Steering Committee statistician, the protocol was amended to analyse only those completing at least 12 weeks on therapy, as this will determine whether the strata result in differences in response (we cannot adequately measure glycaemic response by HbA1c if the patient has been on the drug for less than 12 weeks). A separate analysis will be performed to determine whether the strata influence tolerability by assessing whether the proportion completing at least 12 weeks on therapy differs by drug and strata.
**SA12** v8 20.03.20	Amendment to ensure ongoing participant safety and study integrity during Covid-19 pandemic. Urgent safety measures included (i) extension of visit windows to 14-18 weeks to allow greater flexibility for participants who are unwell/isolating, (ii) provision for remote visits with sample collection outside the usual research setting, (iii) ensuring participants remained on study therapy when only a remote visit is possible, by allowing an additional ‘continuation’ bottle of the same IMP to be issued, or when no other option, transfer to the next IMP without collection of blood samples.

## Supplementary Material

Supplementary Material

## Figures and Tables

**Table 1:** Characteristics of 525 randomised participants.

Variable	n (%) or Mean +/- SD
Male	383 (73%)
Female	142 (27%)
Age at screening (y)	61.9 +/- 9.5
Age at diagnosis (y)^b^	53.0 +- 9.1
Treatment: Metformin only Metformin + sulphonylurea Metformin total daily dose (mg)	254 (48%) 271 (52%) 1753.1 +/- 457.8
Self-reported ethnicity: White Mixed Asian Black Other Not Stated	495 (94%) 2 (0.4%) 16 (3%) 3 (0.6%) 4 (1%) 5 (1%)
BMI (kg/m^2^) BMI>30 n (%)	31.7 +/- 5.5 307 (58%)
eGFR (mls/min/1.73m2) eGFR>90 n(%)	89.7 +/- 13.8 275 (52%)
HbA1c at screening visit (mmol/mol)^a^	69 (63, 78)

a HbA1c presented as median and interquartile range as heavily skewed due to lower limit of inclusion criteria,

b One missing data point for age at diagnosis.

**Table 2:** Adverse events (AEs) on each of the three study drugs.

	Pioglitazone (n=487; 18 withdrew)		Sitagliptin (n=486; 15 withdrew)		Canagliflozin (n=489; 12 withdrew)		Total	
	Participants	Events	Participants	Events	Participants	Events	Participants^a^	Events
Any adverse event	282	686	265	697	299	761	447	2144
Severity of Adverse event								
Mild	231	532	214	530	256	578	403	1640
Moderate	81	125	77	156	91	169	193	450
Severe	18	29	11	11	11	14	38	54
Relatedness of Adverse Event^b^								
Related	158	346	137	346	195	455	319	1147
Unrelated	189	340	182	351	179	306	372	997
Serious Adverse Event								
Any Unrelated	18	20	13	13	11	12	41	45
Death Unrelated	2	3	1	1	0	0	3	4
Any Related^b^	0	0	0	0	0	0	0	0
Adverse Event associated with discontinuation of study drug within 12 weeks (non-tolerability)	19	53	35	140	38	112	82	305
Related^b^	15	37	25	93	28	75	54	205
Unrelated	9	16	21	47	19	37	28	100
Adverse Event associated with withdrawal of study	10	22	5	9	4	10	19	41
Related^b^	2	5	2	2	3	8	7	15
Unrelated	8	17	3	7	1	2	15	26

a Participants experiencing at least one event in that category, regardless of drug.

b Related defined as “Definitely”, “Probably” or “Possibly” related to the drug. Unrelated defined as “Unrelated” or “Not likely” related. NB participants may have more than one AE spanning across more than one category so may be included in multiple categories.

## Data Availability

To minimize the risk of patient re-identification, de-identified individual patient-level clinical data are available under restricted access. Requests for access to anonymised individual participant data (IPD) and study documents should be made to the corresponding author and will be reviewed by the Peninsula Research Bank Steering Committee. Access to data through the Peninsula Research Bank will be granted for requests with scientifically valid questions by academic teams with the necessary skills appropriate for the research. Data that can be shared will be released with the relevant transfer agreement.

## Code availability

Requests for access to code should be made to the corresponding author and will be reviewed by the Peninsula Research Bank Steering Committee Access to code through the Peninsula Research Bank will be granted for requests with scientifically valid questions by academic teams with the necessary skills appropriate for the research. Code will be released with by the lead statistician.
