# Supplementary material for: Patient stratification for determining optimal second and third line therapy for type 2 diabetes: the TriMaster study
Source: Nat Med. Author manuscript; Available in PMC 2023 Feb 22. (PMC7614216; doi:10.1038/s41591-022-02120-7)
Supplement: Supplementary Material [file EMS157312-supplement-Supplementary_Material.pdf]

Table of Contents

Supplementary Tables 2
Supplementary Table 1: Analysis of period and carryover effects 2
Supplementary Table 2: Overall differences in on-treatment characteristics split by drug. 2
Supplementary Table 3: Participant Characteristics Hypothesis 1 3
Supplementary Table 4: Participant Characteristics split by BMI strata 4
Supplementary Table 5: Primary Analysis Hypothesis 1 full mixed effects model 4
Supplementary Table 6: Participant Characteristics Hypothesis 2 5
Supplementary Table 7: Participant Characteristics split by eGFR strata 6
Supplementary Table 8: Primary Analysis Hypothesis 2 full mixed effects model 6
Supplementary Table 9: Sensitivity analysis 1. 7
Supplementary Table 10: Sensitivity analysis 2 7
Supplementary Table 11: Sensitivity analysis 3. 8
Supplementary Table 12 – Tolerability & BMI Strata full mixed effects model 8
Supplementary Table 13: Tolerability sensitivity analysis 9
Supplementary Table 14 – Tolerability & eGFR Strata full mixed effects model 10
Supplementary Table 15: Side effects by BMI Strata – mixed effects models 10
Supplementary Table 16: Side effects by eGFR Strata – mixed effects models 11
Supplementary Table 17: Assessment of period and carryover effect for weight 11
Supplementary Table 18: Hypoglycaemia by BMI strata – mixed effects models 11
Supplementary Table 19: Hypoglycaemia by eGFR strata – mixed effects models 12
Supplementary Table 20: The 24 recruiting centres participating in TriMaster 13
Supplementary Table 21: Side effects in TriMaster 13
Supplementary Information - Acknowledgements 14

Supplementary Tables

Supplementary Table 1: Analysis of period and carryover effects

Output from mixed effects analysis assessing period and carryover effects. Coef represents the coefficient (difference in HbA1c (mmol/mol) between the category and reference category ('ref')), 95% CI is the corresponding 95% confidence interval, z is the z statistic and p is p the value, from a mixed effects model with drug, period, and a carryover variable (i.e. drug in previous period) as fixed effects, participant as a random effect, and HbA1c as the outcome. The global p is the overall p value for comparing across all 3 groups from the same model.

	Coef	95% CI	z	p	Global p
Period (ref=1)					
2	-1.38	-2.54, -0.23	-2.35	0.02	0.02
3	-0.28	-1.46, 0.90	-0.47	0.6	
Carryover (ref=A)					
B	0.98	-0.32, 2.29	1.47	0.14	0.3
C	0.13	-1.19, 1.44	0.19	0.9	

Supplementary Table 2: Overall differences in on-treatment characteristics split by drug.

Differences in on-treatment characteristics for all participants trying therapy (excluding withdrawals). Data presented as mean or n (%) plus 95% confidence intervals in square brackets. P values obtained by mixed effects models with drug (3 level factor) and period (3 level factor) as fixed effects and participant ID as the random effect.

	Treatment			
	Pioglitazone	Sitagliptin	Canagliflozin	p
HbA1c (mmol/mol)[a]	59.6 [58.5, 60.7] n=421	60.0 [59.0, 61.1] n=391	60.6 [59.7, 61.6] n=408	0.2
Discontinued therapy within 12 weeks	26/469 (5.5% [3.7, 8.0])	44/474 (9.3% [6.8, 12.3)	41/474 (8.6% [6.3, 11.6])	0.05
Mean number of side effects	1.49 [1.36, 1.62] n=469	1.19 [1.06, 1.32] n=474	1.58 [1.43, 1.72] n=474	5×10^{-6}
Weight (kg)	95.0 [93.3, 96.7] n=462	93.3 [91.6, 95.0] N=467	91.1 [89.4, 92.8] n=470	8×10^{-171}

Supplementary Table 3: Participant Characteristics Hypothesis 1

Comparison of characteristics between participants with and without valid HbA1cs eligible for hypothesis 1. Data presented as mean +/-SD (with p value assessed using t tests) or n(%) (with p value assessed using chi-squared tests). *HbA1c heavily skewed at baseline due to lower limit of inclusion criteria so median and inter-quartile range presented an p value calculated by Mann-Whitney.

**Variable**	Eligible for Hypothesis 1 (n=356)	Not eligible for hypothesis 1 (n=169)	p
Male	251 (73.3%)	122 (72.2%)	0.07
Age at screening (y)	62.5 +/- 9.1	60.0 +/- 10.2	0.03
Age at diagnosis (y)	53.3 +/-8.9	52.6 +/- 9.4	0.4
Treatment:			
Metformin only	162 (45.5%)	92 (54.4%)	0.056
Metformin + SU	194 (54.5%)	77 (45.6%)	
Ethnicity:			
White	339 (95.2%)	156 (92.3%)	0.2
Mixed	0 (0%)	2 (1.2%)	
Asian	8 (2.3%)	8 (4.7%)	
Black	2 (0.6%)	1 (0.6%)	
Other	4 (1.1%)	0	
Not Stated	3 (0.8%)	2 (1.2%)	
BMI (kg/m^2)	31.8 +/- 5.3	31.5 +/- 5.9	0.7
Obese (BMI>30) n (%)	215 (60.4%)	92 (54.4%)	0.2
eGFR (mls/min/1.73m^2)	89.2 +/- 13.6	90.8 +/- 14.2	0.2
eGFR>90 n(%)	91 (53.9%)	184 (51.7%)	
HbA1c at screening visit (mmol/mol)*	69 (63, 77)	70 (64, 80)	0.04

Supplementary Table 4: Participant Characteristics split by BMI strata

Comparison of baseline characteristics between participants split by BMU strata (BMI<=30 and BMI>30) eligible for hypothesis 1. Data presented as mean +/-SD (with p value assessed using t tests) or n(%) (with p value assessed using Chi-squared tests). *Median and IQR presented for HbA1c (and p value assessed using Mann Whitney) as with other baseline analyses.

**Variable**	BMI≤=30<br>n=141	BMI>30<br>n=215	p
Male	104 (74%)	157 (73%)	0.8
Age at screening (y)	64.2 +/- 8.7	61.5 +/- 9.2	0.006
Age at diagnosis (y)	54.1 +/- 9.4	52.7 +/- 8.6	0.15
Treatment:			
Metformin only	74 (52%)	88 (41%)	0.03
Metformin + SU	67 (48%)	127 (59%)	
Ethnicity:			
White	131 (92.9%)	208 (96.7%)	0.5
Mixed	0	0	
Asian	5 (3.6%)	3 (1.4%)	
Black	1 (0.7%)	1 (0.5%)	
Other	2 (1.4%)	2 (0.9%)	
Not Stated	2 (1.4%)	1 (0.5%)	
eGFR (mls/min/1.73m^2)	87.9 +/-12.7	90.0 +/- 14.1	0.14
HbA1c at screening visit (mmol/mol)*	66 (63, 74)	70 (64, 78)	0.006

Supplementary Table 5: Primary Analysis Hypothesis 1 full mixed effects model

Coef is the regression coefficient, 95% CI is the 95% confidence interval around the coefficient, z is the z statistic, and p is the p value from a mixed effect model with HbA1c as the outcome, participant as the random effect, and drug, period, BMI strata and drug*BMI strata interaction as fixed effects. The drug*BMI strata interaction represents the effect size of interest.

	Coef	95% CI	z	p
Drug (ref=Pioglitazone)				
Sitagliptin	-1.46	-2.94, 0.02	-1.9	0.05
Period (ref=1)				
2	-1.50	-2.77, -0.22	-2.30	0.02
3	-0.07	-1.32, 1.18	-0.11	0.9
BMI>30kg/m^2	-0.68	-2.93, 1.57	-0.6	0.6
**Sitagliptin*BMI>30 interaction**	**2.88**	**0.98, 4.79**	**3.0**	**0.003**

Supplementary Table 6: Participant Characteristics Hypothesis 2

Comparison of characteristics between participants with and without valid HbA1cs eligible for hypothesis 2. Data presented as mean +/-SD (with p value assessed by t tests) or n(%) (with p value assessed by Chi-squared test). *HbA1c heavily skewed at baseline due to lower limit of inclusion criteria so median and inter-quartile range presented an p value calculated by Mann-Whitney.

**Variable**	Eligible for Hypothesis 1 (n=342)	Not eligible for hypothesis 1 (n=183)	p
Male	252 (73.7%)	131 (71.6%)	0.3
Age at screening (y)	62.3 +/- 9.4	61.2 +/- 9.7	0.2
Age at diagnosis (y)	53.2 +/- 9.1	52.7 +/- 9.1	0.6
Treatment:			
Metformin only	162 (47.4%)	92 (50.3%)	0.4
Metformin + SU	180 (52.6%)	91 (49.7%)	
Ethnicity:			
White	326 (95.3%)	169 (92.4%)	0.2
Mixed	0	2 (1.1%)	
Asian	8 (2.3%)	8 (4.4%)	
Black	1 (0.3%)	2 (1.1%)	
Other	4 (1.1%)	0	
Not Stated	5 (1.0%)	2 (1.1%)	
BMI (kg/m^2)	31.7 +/- 5.5	31.6 +/- 5.7	0.7
Obese (BMI>30) n (%)	205 (59.9%)	102 (55.7%)	0.9
eGFR (mls/min/1.73m2)	89.7 +/- 13.9	89.8 +/- 13.5	0.9
eGFR>90 n(%)	179 (53.3%)	96 (52.5%)	0.98
HbA1c at screening visit (mmol/mol)*	69 (63, 77)	70 (64, 79)	0.3

Supplementary Table 7: Participant Characteristics split by eGFR strata

Comparison of baseline characteristics between participants split by eGFR strata (eGFR 60-90 and eGFR>90) eligible for hypothesis 2. Data presented as mean +/-SD (with p value assessed by t tests) or n(%) (with p value assessed by Chi-squared test). *Median and IQR presented for HbA1c (and p value assessed using Mann Whitney) as with other baseline analyses.

**Variable**	eGFR 60-90<br>n=163	eGFR >90<br>n=179	p
Male	127 (77.9%)	125 (69.8%)	0.09
Age at screening (y)	67.2 +/- 7.4	57.8 +/- 8.7	2×10^{-23}
Age at diagnosis (y)	56.9 +/- 7.7	49.8 +/- 8.9	7×10^{-14}
Treatment:<br>Metformin only<br>Metformin + SU	 70 (43%)<br>93 (57%)	 92 (51%)<br>87 (49%)	 0.12
Ethnicity:<br>White<br>Mixed<br>Asian<br>Black<br>Other<br>Not Stated	 160 (98.2%)<br>0<br>3 (1.8%)<br>0<br>0<br>0	 166 (92.7%)<br>0<br>5 (2.8%)<br>1 (0.6%)<br>4 (2.2%)<br>3 (1.7%)	 0.16
BMI (kg/m^2)	31.2 +/- 4.6	32.2 +/- 5.9	0.09
HbA1c at screening visit (mmol/mol)*	68 (62, 77)	70 (64, 78)	0.053

Supplementary Table 8: Primary Analysis Hypothesis 2 full mixed effects model

Coef is the regression coefficient, 95% CI is the 95% confidence interval around the coefficient, z is the z statistic, and p is the p value from a mixed effect model with HbA1c as the outcome, participant as the random effect, and drug, period, eGFR stratum and drug*eGFR strata interaction as fixed effects. The drug*eGFR strata interaction represents the effect size of interest.

	Coef	95% CI	z	P
Drug (ref=Sitagliptin)<br>Canagliflozin	 1.79	 0.55, 3.03	 2.8	 0.005
Period (ref=1)<br>2<br>3	 -1.16<br>-0.15	 -2.33, 0.02<br>-1.33, 1.02	 -1.9<br>-0.3	 0.053<br>0.8
eGFR >90	1.69	-0.41, 3.79	1.6	0.12
**Canagliflozin*eGFR>90 interaction**	**-2.90**	**-4.61, -1.19**	**-3.3**	**0.001**

Supplementary Table 9: Sensitivity analysis 1. Coefficients (and their associated p values) from mixed effects models for each of the drug comparisons for hypothesis 1 and hypothesis 2 adjusted for study epoch (before/after inclusion criteria change to allow metformin only as well as metformin plus SU). Epoch coefficient represents the difference in HbA1c before and after the change in inclusion criteria. Effect size represents the drug*strata interaction from mixed effects models.

		Epoch coef in model [95% CI] (p value)	Main primary analysis effect size [95% CI]	Epoch adjusted analysis effect size [95% CI]
Hypothesis 1 (BMI strata)	Pio v Sit	-2.30 [-5.01, 0.41] (p=0.10)	2.88 [0.98, 4.79]	2.89 [0.98, 4.79]
Hypothesis 2 (eGFR strata)	Sit v Can	-0.87 [-3.47, 1.72] (p=0.5)	-2.90 [-4.61, -1.19]	-2.90 [-4.61, -1.19]

Supplementary Table 10: Sensitivity analysis 2 – comparison of the main effect sizes from the main primary analysis with two sensitivity analyses: a) including only those who were on therapy for at least 15 weeks and b) carrying out adjustment for differences in period intervals between HbA1c measurements. Effect size (and 95% confidence intervals and p values) represents the drug*strata interaction from mixed effects models.

	Main primary analysis: Effect size [95% CI] (p)	a) Sensitivity analysis Effect size [95% CI] (p)	b) Sensitivity analysis Effect size [95% CI] (p)
Hypothesis 1 (BMI strata) Pio v Sit	2.88 [0.98, 4.79]; p=0.003 n=356	2.74 [0.73, 4.76]; p=0.008 n=323	2.62 [0.77, 4.46]; p=0.005 n=356
Hypothesis 2 (eGFR strata) Sit v Can	-2.90 [-4.61, -1.19]; p=0.001 n=342	-3.13 [-4.96, -1.31]; p=0.001 n=307	-2.87 [-4.58, -1.16]; P=0.001 n=342

Supplementary Table 11: Sensitivity analysis 3. Results from mixed effects models of an analysis examining the impact of being on the study drug for >18 weeks (protocol change in light of COVID-19 pandemic). Column 2 is the coefficient (and its corresponding 95% confidence interval and p value) from this mixed effects model which represents the difference in HbA1c between those who had >18 weeks between study visits and those who did not. Effect size represents the drug*strata interaction from mixed effects models.

Comparison	">18 weeks" coef in model [95% CI]; p value	Main primary analysis effect size [95% CI]	">18wk" adjusted sensitivity analysis effect size [95% CI]
Hypothesis 1 (BMI strata) Pio v Sit	-0.84 [-4.56, 2.87]; p=0.7	2.88 [0.98, 4.79]	2.90 [1.00, 4.81]
Hypothesis 2 (eGFR strata) Sit v Can	0.72 [-2.99, 4.43]; p=0.7	-2.90 [-4.61, -1.19]	-2.86 [-4.58, -1.14]

Supplementary Table 12 – Tolerability & BMI Strata full mixed effects model

Results from mixed effects logistic regression for tolerability comparing pioglitazone and sitagliptin across BMI strata. Data presented as odds ratio (OR) for tolerating therapy, 95% confidence intervals, z values and p values from a mixed effects model with participant as the random effect, and drug (reference category pioglitazone), period (reference category 1), BMI strata and drug*BMI strata interaction as fixed effects. (n=943 observations for 480 individuals). As in primary analysis, the key coefficient of interest in each analysis is the drug*strata interaction, representing the difference in odds ratio for tolerating therapy in one drug compared with another for obese v non-obese individuals.

	OR	95% CI	z	p
Drug (ref=sitagliptin)<br>Pioglitazone	1.403	0.634, 3.105	0.84	0.4
Period (ref=1)				
2	0.652	0.306, 1.390	-1.11	0.3
3	0.505	0.239, 1.070	-1.78	0.08
BMI>30kg/m^2	1.382	0.613, 3.115	0.78	0.4
**Pioglitazone*BMI>30 interaction**	**2.11**	**0.657, 6.761**	**1.25**	**0.2**

Supplementary Table 13: Tolerability sensitivity analysis

Comparison of the effect size (drug*strata interaction terms, with their corresponding 95% confidence intervals and p values) from mixed effects models examining tolerability for the two strata for each of the drug pairwise comparisons for the main analysis with tolerability defined as being on therapy for at least 12 weeks, and the sensitivity analysis where tolerhability was defined as being on therapy for at least 15 weeks.

	Main analysis effect size (tolerability >=12wks): OR for tolerability [95% CI] (p)	Sensitivity analysis effect size: (tolerability >=15wks) OR for tolerability [95% CI] (p)
Pio v Sit by BMI strata (Hypothesis 1)	0.47 [0.15, 1.52] (p=0.2)	0.95 [0.42, 2.20] (p=0.9)
Sit v Can by eGFR strata (Hypothesis 2)	0.42 [0.16, 1.14] (p=0.09)	0.38 [0.18, 0.80] (p=0.01)

Supplementary Table 14 – Tolerability & eGFR Strata full mixed effects model

Results from mixed effects logistic regression for tolerability comparing canagliflozin and sitagliptin across eGFR strata. Data presented as odds ratio (OR) for tolerating therapy, 95% confidence intervals, z values and p values from the mixed effects model, with participant as the random effect, and drug (reference category sitagliptin), period (reference category 1), eGFR strata and drug*eGFR strata interaction as fixed effects. (n=948 observations for 487 individuals). As in primary analysis, the key coefficient of interest in each analysis is the drug*eGFR strata interaction, representing the difference in odds ratio for tolerating therapy in one drug compared with another for eGFR60-90 v eGFR>90 individuals.

	OR	95% CI	z	p
Drug (ref=sitagliptin)<br>C	1.755	0.849, 3.629	1.5	0.13
Period (ref=1)<br>2<br>3	0.716<br>1.060	0.392, 1.308<br>0.563, 1.995	-1.09<br>0.18	0.3<br>0.9
eGFR>90	1.408	0.685, 2.900	0.93	0.35
**Canagliflozin*eGFR>90 interaction**	**0.424**	**0.158, 1.135**	**-1.71**	**0.09**

Supplementary Table 15: Side effects by BMI Strata – mixed effects models

Mixed effects logisitic regression model for the odds of experiencing at least one side effect for hypothesis 1 (pioglitazone v sitagliptin by BMI strata). Data presented as odds ratio (OR) for experiencing at least one side effect, 95% confidence intervals, z values and p values from mixed effects model with participant as the random effect, and drug (reference category pioglitazone), period (reference category 1), BMI strata, and drug* BMI strata interaction as fixed effects. (n=943 observations for 480 individuals). As in primary analysis, the key coefficient of interest is the drug*strata interaction

	OR	95% CI	z	p
Drug (ref=pioglitazone)<br>Sitagliptin	 0.719	 0.408, 1.266	 -1.14	 0.3
Period (ref=1)<br>2<br>3	 1.578<br>1.231	 0.963, 2.587<br>0.765, 1.981	 1.81<br>0.86	 0.07<br>0.4
BMI>30kg/m^2	1.882	0.973, 3.640	1.88	0.06
**Sitagliptin*BMI>30 interaction**	**0.676**	**0.313, 1.454**	**-1.0**	**0.3**

Supplementary Table 16: Side effects by eGFR Strata – mixed effects models

Mixed effects logistic regression model for the odds of experiencing at least one side effect for hypothesis 2 (sitagliptin v canagliflozin by eGFR strata). Data presented as odds ratio (OR) for experiencing at least one side effect, 95% confidence intervals, z values and p values from mixed effects models with participant as a random effect, and drug (reference category sitagliptin), period (reference category 1), eGFR strata and drug*eGFR strata interaction as fixed effects. (n=943 observations for 480 individuals). As in primary analysis, the key coefficient of interest is the drug*strata interaction

	OR	95% CI	z	p
Drug (ref=sitagliptin)				
Canagliflozin	1.328	0.791, 2.230	1.1	0.3
Period (ref=1)				
2	1.188	0.738, 1.911	0.7	0.5
3	0.742	0.469, 1.175	-1.3	0.2
eGFR>90	1.038	0.600, 1.807	0.1	0.9
**Canagliflozin*eGFR>90 interaction**	**1.461**	**0.702, 3.040**	**1.0**	**0.3**

Supplementary Table 17: Assessment of period and carryover effect for weight

Output from mixed effects analysis assessing period and carryover effects for weight. Coef represents the coefficient (difference in weight (kg) between the category and reference category ('ref')), 95% CI is the corresponding 95% confidence interval, z is the z statistic and p is p the value, from a mixed effects model with drug, period, and a carryover variable (i.e. drug in previous period) as fixed effects, participant as a random effect, and weight (kg) as the outcome.

Variable	Coef (95% CI)	Z	P
Drug:			
Pioglitazone (ref)			
Sitagliptin	-1.86 (-2.15, -1.57)	-12.6	2×10^{-36}
Canagliflozin	-4.23 (-4.52, -3.94)	-28.5	9×10^{-179}
Period:			
1 (ref)			
2	0.94 (0.60, 1.28)	5.4	6×10^{-8}
3	1.03 (0.68, 1.37)	5.8	6×10^{-9}
Carryover (drug in previous period)			
Pioglitazone (ref)			
Sitagliptin	-0.94 (-1.33, -0.55)	-4.7	2×10^{-6}
Canagliflozin	-1.37 (-1.75, -0.98)	-6.9	7×10^{-12}

Supplementary Table 18: Hypoglycaemia by BMI strata – mixed effects models

Mixed effects logistic regression model for the odds of experiencing hypoglycaemia for hypothesis 1 (pioglitazone v sitagliptin by BMI strata). Data presented as odds ratio (OR) for experiencing hypoglycaemia, 95% confidence intervals, z values and p values for a mixed effects model with participant as a random effect and drug (reference category pioglitazone), period (reference category 1), BMI strata, and drug* BMI strata interaction as fixed effects. (n=902 observations for 480 individuals). As in primary analysis, the key coefficient of interest is the drug*strata interaction

	OR	95% CI	z	p
Drug (ref=pioglitazone)<br>Sitagliptin	0.992	0.413, 2.379	-0.02	0.99
Period (ref=1)<br>2<br>3	 0.817<br>0.465	 0.428, 1.560<br>0.232, 0.931	 -0.6<br>-2.2	 0.5<br>0.03
BMI>30kg/m^2	1.71	0.698, 4.185	1.17	0.2
**Sitagliptin*BMI>30 interaction**	**0.744**	**0.249, 2.216**	**-0.5**	**0.6**

Supplementary Table 19: Hypoglycaemia by eGFR strata – mixed effects models

Mixed effects logistic regression model for the odds of experiencing hypoglycaemia for hypothesis 2 (sitagliptin v canagliflozin by eGFR strata). Data presented as odds ratio (OR) for experiencing hypoglycaemia, 95% confidence intervals, z values and p values for a mixed effects model with participant as a random effect and drug (reference category sitagliptin), period (reference category 1), eGFR strata and drug*eGFR strata interaction as fixed effects. (n=921 observations for 485 individuals). As in primary analysis, the key coefficient of interest is the drug*strata interaction

	OR	95% CI	z	p
Drug (ref=sitagliptin)<br>Canagliflozin	0.522	0.192, 1.420	-1.3	0.2
Period (ref=1)<br>2<br>3	 0.482<br>0.725	 0.200, 1.162<br>0.318, 1.652	 -1.6<br>-0.8	 0.1<br>0.4
eGFR>90	2.164	0.745, 6.286	1.4	0.2
**Canagliflozin*eGFR>90 interaction**	**0.730**	**0.199, 2.672**	**-0.5**	**0.6**

Supplementary Table 20: The 24 recruiting centres participating in TriMaster

1	Royal Devon and Exeter NHS Foundation Trust - RD&E Wonford Hospital
2	NHS Tayside - Ninewells Hospital & Medical School
3	Greater Glasgow & Clyde Health Board - BHF Glasgow Cardiovascular Research Centre
4	Taunton and Somerset NHS Foundation Trust - Musgrove Park Hospital
5	Brighton and Sussex University Hospitals NHS Trust - Royal Sussex County Hospital
6	Cumbria Partnership NHS Foundation Trust - Cumberland Infirmary
7	Oxford University Hospitals - Churchill Hospital
8	Sheffield Teaching Hospitals NHS Foundation Trust - Northern General Hospital
9	The Newcastle Upon Tyne Hospitals NHS Foundation Trust - Royal Victoria Infirmary
10	Barnsley Hospital NHS Foundation Trust - Barnsley Hospital
11	North Bristol NHS Trust - Southmead Hospital
12	Plymouth Hospitals NHS Trust - Derriford Hospital
13	Hywel Dda University Health Board - Prince Philip Hospital
14	Abertawe Bro Morgannwg University Health Board – Morriston Hospital
15	Royal Cornwall Hospitals NHS Trust - Royal Cornwall Hospital
16	Cardiff and Vale University Health Board - University Hospital of Wales
17	Guy's and St Thomas' NHS Foundation Trust - Guy's Hospital
18	Surrey and Sussex Healthcare NHS Trust - East Surrey Hospital
19	East Kent Hospitals University NHS Foundation Trust - QETQM Hospital
20	Harrogate and District NHS Foundation Trust - Harrogate District Hospital
21	Buckinghamshire Healthcare NHS Trust - Wycombe General Hospital
22	East Suffolk and North Essex NHS Foundation Trust - Ipswich Hospital
23	Royal Berkshire NHS Foundation Trust - Royal Berkshire Hospital
24	Portsmouth Hospitals NHS Trust - Queen Alexandra Hospital

Supplementary Table 21: Side effects in TriMaster

Side effects asked about at baseline and follow-up study visits:

Swollen ankles/legs
Weight gain
Broken bones
Low blood sugar
Headache
Increased thirst
Passing more urine
Feeling dehydrated
Tiredness
Difficulty sleeping
Rashes
Change in appetite with weight change
Feeling or being sick
Constipation / diarrhoea
Urine infection
Thrush / Rash or redness of foreskin

Supplementary Information - Acknowledgements

TriMaster Central Coordinating Team: Catherine Angwin, Dr Caroline Jenkinson, Nina Rickards, Claire Thorne

TriMaster Study Group: Andrew Hattersley (PI), Catherine Angwin, Claire Ball, Anna Barnes, Tamika Chapter, Daniela Carmona, Tim Cranston, Clare Davidson, Mary Davis, Evan Davy, Tim Eames, Joanne Findlay, Diane Jarvis, Caroline Jenkinson, Angus Jones, John Kirkwood, Bethan Knight, Bridget Knight, James Leavy, Pattie Liakos, Kelly Littlewood, Timothy McDonald, Dionne McGill, Richard Oram, Nicola Pamphilon, Kashap Patel, Andrew Pitt, Lynne Quinn, Shelley Rhodes, Nina Rickards, Emma Robjohns, Kim Rowden, Sofia Sanabria, Beverley Shields, Steven Spaull, Sarah Statton, Anna Steele, Nick Thomas, Claire Thorne, Shirley Todd, Harry Tripp, Robert Wells, Luke Weymouth, Fiona Walters, Ewan Pearson (PI), Gill Reekie, Charlene Wong, Naveed Sattar (PI), Josephine Conney, Robert Lyndsay, Kirsty McLeish, Janice Richardson, Rob Andrews (PI), Ian Bodger, Richard Burgess, Sue Crouch, Isy Douek, Amanda Groves, Catherine Lane, Claire Lorimer, Joy Rowe, Lyndsay Stone, Ali Chakera (PI), Zdenka Cipinova, Zhengmai He, Allison Leslis, Dominika Wlazly, Louise Overend (PI), Sushil Kumar, Joanne Rafferty, Gillian Webster, Rustam Rea (PI), Lia Anguelova Karyna Gibbons, Nicky McRobert, Ursula Taylor, Simon Heller (PI), Mark Davy, Jackie Elliot, Rajiv Ghandi, Sue Hudson, Linda Greaves, Chloe Husband, Peter Novodorsky, Helena Renberg-Fawcet, Kim Ryalls, Lisa Zeidan, Mark Walker (PI), Ahmad AbuSaleh, Jade Davison, Margaret Fearby, Louise Finlay, Donna McEvoy, Ian McVittie, Bijal Patel, Geraldine Richel,l Rebecca Wilson, Thomas Hugh Jones (PI), Mishell Cunningham, Allison Daniels, Laura Walker, Lisa Zeidan, Andrew Johnson (PI), Georgina Russell (PI), Jade Bennett, Shenede Coppin, Joanne Davies, Sharon Hook, Abby Hookey, Jade King, Sharon Merritt, Helen Pearson, Sharon Tovey, Jill Townley, Patrick English (PI), Migaila Aldred, Emma Bishop, Emma Storr, Sam Rice (PI), Kim Davies, Rachel Davies, Linda O'Brien, Zohra Omar, Stephen Bain (PI), Lucy Barlow, Steven Creely (PI), Duncan Browne, Terri Chant, Helen Chenoweth, Kelly Hutchins, Laura Jones, Haider Khan, Emily Laity, Hemanth Bolusani (PI), Adele Farrugia, Grace Hopkins, Emma Norling, Caroline Robinson, Kennedy Cruickshank (PI), Krzystof Rutkowski, Benjamin Field (PI), James Clark, Ed Combes, Ruth Habibi, Stonny Joseph (PI), Louise Allen, Tracy Hazleton, Alicia Knight, Janine Musselwhite, Sutapa Ray (PI), Amanda Gillespie, Christine Cassidy, Peter Hammond, Tahir Idrees, Sam Jackson Neil Lauber, Deirdre Maguire, Ayeaye Min, Simon Trickett, Taryn Ward, Annie Williamson, Chitrabhanu Ballav (PI), Nicola Bowers, Sonia Dayal, Flavio Gil Lopes de Sousa, Lisa Jones, Mari Kononen, Ruth Penn, Fabiana Saraiva, El Muhtadi Saeed (PI), Julie Sutton, Gerry Rayman (PI), Helen Atkins, Emma Galloway, Jo Rosier, Debbie Simmonds, Michael Cummings (PI), Katherine Alington Sharon Allard, Sophie Daltry, Christine Hall, Victoria Hunter, Kate Millar.

MASTERMIND Consortium (additional members): Prof Michael Weedon, Dr Robert Lindsay, Prof Christopher Jennison, Prof Mark Walker, Prof Kennedy Cruickshank, Dr Salim Janmohamed, Prof Christopher Hyde, Prof Alastair Gray, Prof Stephen Gough, Dr Lauren Rodgers, Prof Timothy McDonald, Dr Olorunsola Agbaje

TriMaster Data Monitoring Committee: Prof David Owens (Chair), Prof Kamlesh Khunti and Prof Christopher Weir

TriMaster Trial Steering Committee: Prof Edwin Gale (Chair), Prof Polly Bingley, Caroline Padget, Prof David Russell-Jones, and Prof Stephen Senn
